# Supplementary material for: Treatment response to bulevirtide is linked to amelioration of portal hypertension in patients with chronic hepatitis D
Source: JHEP Rep. 2025 Oct 17;8(1):101643. doi: 10.1016/j.jhepr.2025.101643 (PMC12890450; doi:10.1016/j.jhepr.2025.101643)
Supplement: Multimedia component 1 [file mmc1.pdf]

Treatment response to bulevirtide is linked to amelioration of portal hypertension in patients with chronic hepatitis D☆

Lisa Sandmann, Mathias Jachs, Tammo L. Tergast, Lukas Hartl, Birgit Bremer, Martin A. Kabelitz, Michael Schwarz, Julius F.M. Egge, Lorenz Balcar, Benedikt Silvester Hofer, Christine S. Falk, Albert Friedrich Stättermayer, Markus Cornberg, Michael Trauner, Katja Deterding, Mattias Mandorfer, Heiner Wedemeyer, Thomas Reiberger, Benjamin Maasoumy

Table of contents

Supplementary methods 2

Fig. S1 3

Table S1. 4

Table S2. 5

Table S3. 6

Table S4. 7

Table S5. 8

Table S6. 12

Table S7. 15

Table S8. 17

Supplementary methods

Exploratory analysis of PH-related biomarkers and systemic inflammatory markers

The following ELISAs were used according to the manufacturers' instructions: LBP (ab213805, Abcam, Cambridge, UK), CD163 (Quantikine® ELISA Human CD163 Immunoassay, R&D Systems, Catalog Number DC 1630), Ang1 (Quantikine® ELISA Human Angiopoietin-1 Immunoassay, R&D Systems, Catalog Number DANG10), Ang2 (Quantikine® ELISA Human Angiopoietin-2 Immunoassay, R&D Systems, Catalog Number DANG20), and TGFb (Quantikine® ELISA Human TGF-β1 Immunoassay, R&D Systems, Catalog Number DB100C).

Supplementary figures

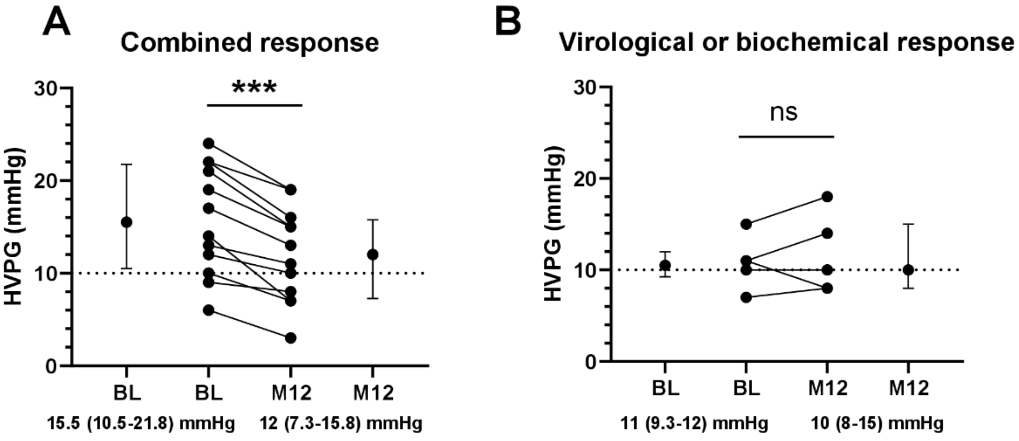

Supplementary figure 1: Comparison of HVPG at baseline and M12 of patients with combined (A) or with virological/biochemical (B) response. Medians with interquartile range and individual values are depicted. Wilcoxon signed rank test was used to compare baseline and M12 results.

*** $p < 0.001$; ns, not significant

Table S1.

HVPG (mmHg)		Responder	Non-responder	p-value
CR	BL	15.5 (10.5-21.8)	11 (10-14)	0.129
	M12	12 (7.3-15.8)	10 (8.5-17)	0.923
	Median HVPG change (BL-M12) [mmHg]	-3.5 (-5.75 - -2.25)	0.5 (-0.75 – 3.0)	<0.001
VR	BL	14.5 (10-21.3)	11 (9.3-12.8)	0.172
	M12	12 (7.8-16.5)	10 (8-15.8)	0.828
	Median HVPG change (BL-M12) [mmHg]	-3 (-5.25 - -1.75)	0.5 (-1.5 – 3.0)	0.012
BR	BL	12.5 (10-20.5)	13 (10.3-17.3)	0.950
	M12	10.5 (8-15)	14 (10-20.3)	0.272
	Median HVPG change (BL-M12) [mmHg]	-3.0 (-4.75 - -1.25)	1.5 (-0.75 – 3.0)	0.013

Table S1. Comparison of baseline and on-treatment HVPG values stratified by treatment response. Median with interquartile range for continuous parameters is depicted, Mann Whitney U test was used to compare groups.

Abbreviations: BL, baseline; BR, biochemical response; CR, combined response; HVPG, hepatic venous pressure gradient; M12, minimum of 12 months of antiviral treatment; VR, virological response

Table S2.

LSM (kPa)		Baseline	M12	p-value
All patients		24 (13.5-28.5)	13.5 (9.0-22.7)	**<0.001**
CR	Yes	24.1 (13.9-31.2)	13.3 (9.6-22.1)	0.004
	No	20.8 (11-26)	14.4 (7.3-25)	0.039
VR	Yes	24.1 (15.7-30.7)	16.6 (10-23.9)	0.002
	No	16.2 (9.1-25.5)	10.5 (6.8-19.4)	0.156
BR	Yes	18.7 (12-28)	11.5 (8.8-18.7)	0.001
	No	25.8 (24.3-30)	23.9 (17.9-23.9)	0.250

Table S2. Comparison of baseline and on-treatment liver stiffness measurement results stratified by treatment response. Median with interquartile range for continuous parameters is depicted, Wilcoxon signed rank test was used to compare groups.

Abbreviations: BL, baseline; BR, biochemical response; CR, combined response; LSM, liver stiffness measurement; M12, minimum of 12 months of antiviral treatment; VR, virological response

Table S3.

LSM (kPa)		Responder	Non-responder	p-value
CR	BL	24.1 (13.9-31.2)	20.8 (11-26)	0.473
	M12	13.3 (9.6-22.1)	14.4 (7.3-25)	0.792
VR	BL	24.1 (15.7-30.7)	16.2 (9.1-25.5)	0.179
	M12	16.6 (10-23.9)	10.5 (6.8-19.4)	0.246
BR	BL	18.7 (12-28)	25.8 (24.3-30)	0.152
	M12	11.5 (8.8-18.7)	23.9 (17.9-23.9)	0.052

Table S3. Comparison of baseline and on-treatment liver stiffness measurement values stratified by treatment response. Median with interquartile range for continuous parameters is depicted, Mann Whitney U test was used to compare groups.

Abbreviations: BL, baseline; BR, biochemical response; CR, combined response; LSM, liver stiffness measurement; M12, minimum of 12 months of antiviral treatment; VR, virological response

Table S4.

	BL			M12		
	CSPH “ruled out”		CSPH “ruled in”	CSPH “ruled out”		CSPH “ruled in”
Total cohort: n = 20	LSM ≤ 15 kPa and PLT ≥ 150 G/L	unclassified	LSM ≥ 25 kPa	LSM ≤ 15 kPa and PLT ≥ 150 G/L	unclassified	LSM ≥ 25 kPa
Patients, n (%)	2 (10%)	11 (55%)	7 (35%)	1 (5%)	15 (75%)	4 (20%)
CSPH, n	1	9	7	0	10	4
Performance	NPV 50% Sensitivity 94%		PPV 100% Specificity 100%	NPV 100% Sensitivity 100%		PPV 100% Specificity 100%

Table S4. Diagnostic performance of the Baveno VII criteria at baseline and after a minimum treatment duration of 12 months.

Abbreviations: CSPH, clinically significant portal hypertension; HVPG, hepatic venous pressure gradient; LSM, liver stiffness measurement; M12, minimum of 12 months of antiviral treatment; NIT, non-invasive test

Table S5.

		Baseline	M12	p-value
**LBP (ng/mL)**				
All patients		3477 (2384-4241)	3265 (2108-4472)	0.623
Combined response	Yes	3688 (2939-(4241)	3545 (2513-4638)	0.765
	No	2805 (2060-4683)	2611 (1684-4357)	0.383
Virological response	Yes	3477 (2404-4121)	3265 (2324-4507)	0.735
	No	3477 (2141-5156)	3384 (1699-5271)	0.563
Biochemical response	Yes	3688 (2514-4543)	6545 (2108-4348)	0.847
	No	2805 (1739-3598)	2611 (1510-3682)	0.625
**sCD163 (ng/mL)**				
All patients		**1813 (1315-2063)**	**1405 (1041-1965)**	**0.003**
Combined response	**Yes**	**1865 (1629-2609)**	**1405 (1049-1965)**	**0.001**
	No	1223 (1100-2004)	1367 (1041-1968)	0.313
Virological response	**Yes**	**1865 (1615-2094)**	**1405 (1029-1832)**	**<0.001**
	No	1199 (1077-1934)	1403 (997-2157)	0.688
Biochemical response	**Yes**	**1841 (1587-2154)**	**1405 (1021-1965)**	**<0.001**
	No	1223 (1112-1858)	1367 (1099-2063)	1.0
**TGFb (pg/mL)**				
All patients		18719 (12025-24968)	18587 (14483-23160)	0.324
Combined response	Yes	19926 (16537-24968)	18587 (14596-24569)	0.515
	No	14848 (10585-26292)	18482 (11810-23160)	0.641
Virological response	Yes	18719 (12486-23480)	17584 (14170-23152)	0.903
	No	19509 (9619-28974)	22209 (13677-24931)	0.844
	Yes	18718 (12970-22562)	20117 (14596-23160)	0.528

Biochemical response	No	20214 (11860-31502)	15511 (11810-26598)	1.0
**PDGF (pg/mL)**				
All patients		138.4 (56.4-330.8)	152.9 (46.7-263.9)	0.475
Combined response	Yes	125.5 (51.1-295)	109.6 (28.2-224.2)	0.301
	No	207.6 (72.6-374.6)	210.7 (86.6-278.5)	0.945
Virological response	Yes	115.7 (53.7-264.5)	109.6 (38.2-229.8)	0.542
	No	327.9 (101.1-395.1)	231 (118.9-354.4)	0.844
Biochemical response	Yes	138.4 (51.1-303.2)	143.5 (34.1-262.7)	0.274
	No	249.4 (72.6-435.6)	210.7 (96.6-491.6)	0.875
**Ang1 (pg/mL)**				
All patients		15176 (8272-20352)	14099 (8938-19913)	0.245
Combined response	Yes	15176 (10928-19596)	13074 (9674-19913)	0.233
	No	13218 (6745-20762)	14303 (7116-22836)	0.844
Virological response	Yes	15020 (9256-19171)	12348 (9182-19641)	0.426
	No	19595 (5838-22063)	17065 (7547-24339)	0.563
Biochemical response	Yes	15176 (9942-19596)	15028 (9674-19913)	0.298
	No	14157 (6849-24438)	10568 (7077-21455)	0.875
**Ang2 (pg/mL)**				
All patients		**2793 (1870-3604)**	**2527 (1379-3135)**	**0.030**
Combined response	**Yes**	**3542 (1955-3815)**	**2227 (1424-3377)**	**0.001**
	No	2423 (1581-2925)	2607 (1284-3080)	0.945
Virological response	**Yes**	**3250 (1911-3678)**	**2227 (1462-3142)**	**<0.001**
	No	2423 (1478-3159)	2613 (1197-3997)	0.688
	**Yes**	**3159 (1955-3815)**	**2527 (1424-3135)**	**0.013**

Biochemical response	No	2326 (1541-2925)	2147 (1241-5552)	0.875
**Ang2/Ang1 ratio**				
All patients		0.22 (0.10-0.35)	0.18 (0.08-0.35)	0.179
Combined response	Yes	0.21 (0.10-0.35)	0.18 (0.08-0.32)	0.266
	No	0.22 (0.09-0.41)	0.16 (0.08-0.39)	0.461
Virological response	Yes	0.24 (0.10-0.38)	0.18 (0.09-0.36)	0.104
	No	0.17 (0.07-0.77)	0.16 (0.06-0.47)	0.844
Biochemical response	Yes	0.22 (0.10-0.35)	0.18 (0.08-0.32)	0.106
	No	0.19 (0.08-0.40)	0.26 (0.06-0.73)	0.875
**VEGF (pg/mL)**				
All patients		34.6 (34.6-813.6)	34.6 (34.6-4671.5)	0.074
Combined response	Yes	34.6 (34.6-618.8)	34.6 (34.6-5064.9)	0.313
	No	34.6 (34.6-1661.8)	34.6 (34.6-4242.5)	0.250
Virological response	Yes	34.6 (34.6-2214)	34.6 (34.6-5355.9)	0.219
	No	34.6 (34.6-441.4)	34.6 (34.6-3384)	0.500
Biochemical response	Yes	34.6 (34.6-34.6)	34.6 (34.6-4671.5)	0.156
	No	1661.8 (34.6-5180.8)	1495 (34.6-5885.6)	0.500

Table S5. Comparison of PH-related biomarkers reflecting bacterial translocation, macrophage activation, (dys)angiogenesis and fibrogenesis. Wilcoxon signed rank test was used to compare baseline and M12 parameters. Abbreviations: Ang1, Angiopoietin-1; Ang2, Angiopoietin-2; LBP, lipopolysaccharide-binding protein; M12, minimum of 12 months of antiviral treatment; PDGF, platelet-derived growth factor; TGFb, transforming growth factor beta; VEGF, vascular endothelial growth factor

Table S6.

		Responder	Non-responder	p-value
**LBP (ng/mL)**				
CR	BL	3688 (2939-(4241)	2805 (2060-4683)	0.343
	M12	3545 (2513-4638)	2611 (1684-4357)	0.473
VR	BL	3477 (2404-4121)	3477 (2141-5156)	0.968
	M12	3265 (2324-4507)	3384 (1699-5271)	0.904
BR	BL	3688 (2514-4543)	2805 (1739-3598)	0.178
	M12	6545 (2108-4348)	2611 (1510-3682)	0.335
**sCD163 (ng/mL)**				
CR	BL	1865 (1629-2609)	1223 (1100-2004)	0.069
	M12	1405 (1049-1965)	1367 (1041-1968)	0.910
VR	BL	1865 (1615-2094)	1199 (1077-1934)	0.062
	M12	1405 (1029-1832)	1403 (997-2157)	0.904
BR	BL	1841 (1587-2154)	1223 (1112-1858)	0.178
	M12	1405 (1021-1965)	1367 (1099-2063)	0.820
**TGFb (pg/mL)**				
CR	BL	19926 (16537-24968)	14848 (10585-26292)	0.521
	M12	18587 (14596-24569)	18482 (11810-23160)	0.734
VR	BL	18719 (12486-23480)	19509 (9619-28974)	0.904
	M12	17584 (14170-23152)	22209 (13677-24931)	0.602
BR	BL	18718 (12970-22562)	20214 (11860-31502)	0.617
	M12	20117 (14596-23160)	15511 (11810-26598)	0.554
**PDGF (pg/mL)**				
CR	BL	125.5 (51.1-295)	207.6 (72.6-374.6)	0.521

	M12	109.6 (28.2-224.2)	210.7 (86.6-278.5)	0.176
VR	BL	115.7 (53.7-264.5)	327.9 (101.1-395.1)	0.207
	M12	109.6 (38.2-229.8)	231 (118.9-354.4)	0.173
BR	BL	138.4 (51.1-303.2)	249.4 (72.6-435.6)	0.437
	M12	143.5 (34.1-262.7)	210.7 (96.6-491.6)	0.283
**Ang1 (pg/mL)**				
CR	BL	15176 (10928-19596)	13218 (6745-20762)	0.678
	M12	13074 (9674-19913)	14303 (7116-22836)	0.910
VR	BL	15020 (9256-19171)	19595 (5838-22063)	0.602
	M12	12348 (9182-19641)	17065 (7547-24339)	0.602
BR	BL	15176 (9942-19596)	14157 (6849-24438)	0.936
	M12	15028 (9674-19913)	10568 (7077-21455)	0.494
**Ang2 (pg/mL)**				
CR	BL	3542 (1955-3815)	2423 (1581-2925)	0.246
	M12	2227 (1424-3377)	2607 (1284-3080)	0.910
VR	BL	3250 (1911-3678)	2423 (1478-3159)	0.363
	M12	2227 (1462-3142)	2613 (1197-3997)	1.0
BR	BL	3159 (1955-3815)	2326 (1541-2925)	0.242
	M12	2527 (1424-3135)	2147 (1241-5552)	0.892
**Ang2/Ang 1 ratio**				
CR	BL	0.21 (0.10-0.35)	0.22 (0.09-0.41)	0.970
	M12	0.18 (0.08-0.32)	0.16 (0.08-0.39)	0.970
VR	BL	0.24 (0.10-0.38)	0.17 (0.07-0.77)	0.602
	M12	0.18 (0.09-0.36)	0.16 (0.06-0.47)	0.904
BR	BL	0.22 (0.10-0.35)	0.19 (0.08-0.40)	0.750

	M12	0.18 (0.08-0.32)	0.26 (0.06-0.73)	0.820
**VEGF (pg/mL)**				
CR	BL	34.6 (34.6-618.8)	34.6 (34.6-1661.8)	0.881
	M12	34.6 (34.6-5064.9)	34.6 (34.6-4242.5)	0.900
VR	BL	34.6 (34.6-2214)	34.6 (34.6-441.4)	0.447
	M12	34.6 (34.6-5355.9)	34.6 (34.6-3384)	0.524
BR	BL	34.6 (34.6-34.6)	1661.8 (34.6-5180.8)	0.097
	M12	34.6 (34.6-4671.5)	1495 (34.6-5885.6)	0.645

Table S6. Comparison of baseline and on-treatment biomarkers stratified by treatment response. Median with interquartile range for continuous parameters is depicted, Mann Whitney U test was used to compare groups.

Abbreviations: Ang1, Angiopoietin-1; Ang2, Angiopoietin-2; BL, baseline; BR, biochemical response; CR, combined response; LBP, lipopolysaccharide-binding protein; M12, minimum of 12 months of antiviral treatment; PDGF, platelet-derived growth factor; TGFb, transforming growth factor beta; VEGF, vascular endothelial growth factor; VR, virological response

Table S7.

	HVPG decline ≥10%<br>n = 13		p-value	No or < 10 % HVPG decline<br>n = 7		p-value
	Baseline	M12		Baseline	M12	
LBP (ng/mL)	3779 (3063-4462)	3717 (2593-4854)	0.910	2374 (2020-3719)	2434 (1677-3980)	0.078
sCD163 (ng/mL)	**1874 (1687-2222)**	**1431 (1088-2076)**	**0.001**	1204 (1081-1826)	1290 (1035-1515)	0.469
TGF beta (pg/mL)	19654 (16580-24224)	18966 (14989-24221)	0.735	12717 (10255-27711)	15696 (10639-23149)	0.938
PDGF (pg/mL)	133.3 (53.8-279.7)	80.4 (27.1-219.8)	0.191	282.0 (55.5-374.8)	233.5 (148.2-280.1)	0.813
Ang 1 (pg/mL)	15240 (11331-20323)	14825 (10044-19777)	0.146	7781 (6538-20533)	13373 (6901-24150)	0.938
Ang 2 (pg/mL)	**3551 (1979-4055)**	**2630 (1510-3259)**	**0.001**	2061 (1492-2803)	2424 (1211-3172)	0.578
Ang 2/Ang 1 ratio	0.20 (0.10-0.35)	0.18 (0.08-0.29)	0.216	0.24 (0.08-0.45)	0.13 (0.06-0.41)	0.578
VEGF (pg/mL)	34.6 (34.6-424.1)	34.6 (34.6-3876.6)	0.313	34.6 (34.6-2541.6)	34.6 (34.6-4671.5)	0.250

Table S7. Comparison of markers reflecting bacterial translocation, macrophage activation, neoangiogenesis and fibrogenesis in patients with and without ≥ 10% HVPG decline. Wilcoxon signed rank test was used to compare baseline and M12 parameters.

Abbreviations: Ang1, Angiopoietin-1; Ang2, Angiopoietin-2; LBP, lipopolysaccharide-binding protein; M12, minimum of 12 months of antiviral treatment; PDGF, platelet-derived growth factor; TGFb, transforming growth factor beta; VEGF, vascular endothelial growth factor

Table S8.

		Baseline	M12	p-value
**CRP (mg/l)**				
All patients		1.4 (0.65-2.48)	0.75 (0.6-3.55)	0.396
CR	Yes	2.1 (0.93-3.25)	0.65 (0.6-3.55)	0.140
	No	0.85 (0.6-1.5)	0.85 (0.6-3.83)	0.750
VR	Yes	1.95 (0.75-2.75)	0.65 (0.6-3.45)	0.140
	No	1.05 (0.58-2)	1.05 (0.6-10.4)	0.750
BR	Yes	1.95 (0.93-3.03)	0.8 (0.6-3.55)	0.260
	No	0.7 (0.53-0.88)	0.7 (0.6-3.73)	1.0
**WBC (x1000/µl)**				
All patients		3.6 (2.6-4.2)	3.2 (2.2-4.4)	0.280
CR	Yes	3.55 (2.7-4.3)	3.2 (2.4-3.8)	0.077
	No	3.7 (2.5-4.2)	3.6 (2.1-4.7)	0.570
VR	Yes	3.6 (2.5-4.2)	3.2 (2.2-3.9)	0.093
	Yes	3.7(2.6-4.7)	3.8 (2.2-5.2)	0.688
BR	Yes	3.6 (2.6-4.3)	3.2 (2.4-4.1)	0.298
	No	3.7 (2.3-4.2)	3.2 (1.9-4.6)	1.0

Table S8. Comparison of baseline and on-treatment levels of c-reactive protein and white blood cell counts stratified by treatment response. Median with interquartile range for continuous parameters is depicted, Wilcoxon signed rank test was used to compare groups.

Abbreviations: BL, baseline; BR, biochemical response; CR, combined response; CRP, c-reactive protein; M12, minimum of 12 months of antiviral treatment; VR, virological response; WBC, white blood cells
