# Supplementary material for: Treatment response to bulevirtide is linked to amelioration of portal hypertension in patients with chronic hepatitis D
Source: JHEP Rep. 2025 Oct 17;8(1):101643. doi: 10.1016/j.jhepr.2025.101643 (PMC12890450; doi:10.1016/j.jhepr.2025.101643)
Supplement: Multimedia component 4 [file mmc4.pdf]

JHEP|Reports

Treatment response to bulevirtide is linked to amelioration of portal hypertension in patients with chronic hepatitis D

Authors

Lisa Sandmann, Mathias Jachs, Tammo L. Tergast, …, Heiner Wedemeyer, Thomas Reiberger, Benjamin Maasoumy

Correspondence

sandmann.lisa@mh-hannover.de (L. Sandmann).

Graphical abstract

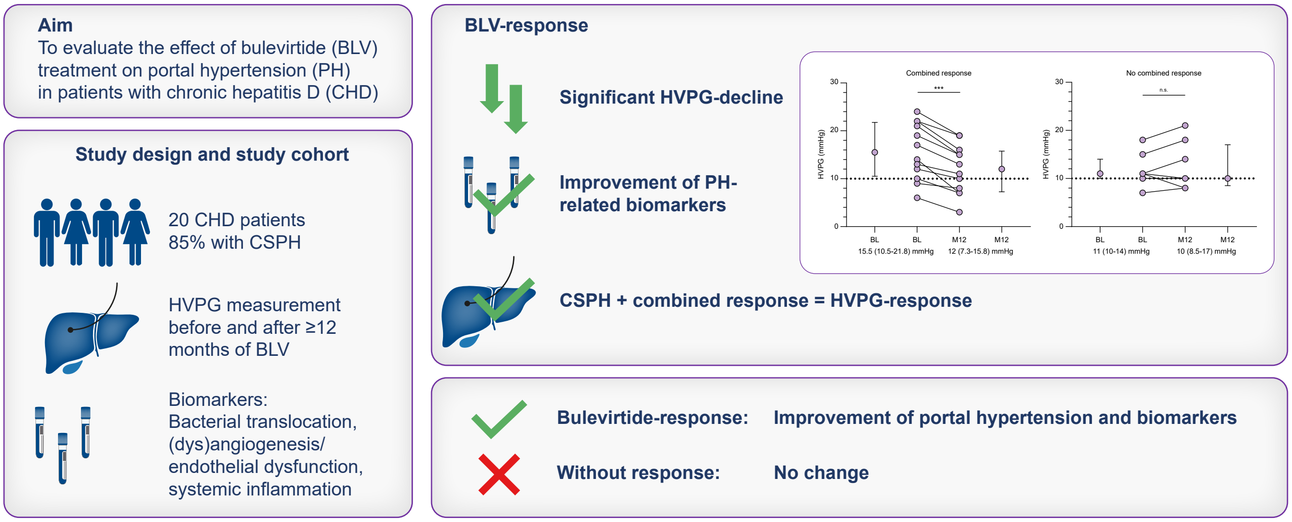

Highlights:

- Response to antiviral treatment with bulevirtide ameliorates portal hypertension as measured by HVPG.
- A clinically meaningful HVPG reduction occurred only in treatment responders as defined by endpoints used in clinical trials.
- All patients with combined response showed a clinically meaningful HVPG response.
- These findings reinforce the clinical relevance of the proposed on-treatment response criteria for bulevirtide therapy.

Impact and implications:

Portal hypertension is the main mechanism driving clinical deterioration in patients with compensated advanced chronic liver disease, for which patients with chronic hepatitis D (CHD) are at particularly high risk. This study demonstrates that in patients with CHD with portal hypertension, achieving response to antiviral treatment with bulevirtide decreases the hepatic venous pressure gradient (HVPG). Importantly, a clinical meaningful reduction in HVPG was observed only in treatment responders as defined by endpoints used in clinical trials, in particular, in all patients achieving combined response. Biomarkers of important pathophysiological mechanisms were also improved. The finding of a clinical meaningful HVPG decline in patients with CHD responding to antiviral treatment strengthens the clinical significance of the suggested on-treatment response criteria, supporting a disease-modifying effect of BLV response, likely translating into decreased morbidity and mortality in patients with CHD.

https://doi.org/10.1016/j.jhepr.2025.101643
© 2025 The Author(s). Published by Elsevier B.V. on behalf of European Association for the Study of the Liver (EASL). This is an open access article under the CC BY license (http://creativecommons.org/licenses/by/4.0/).

Treatment response to bulevirtide is linked to amelioration of portal hypertension in patients with chronic hepatitis D☆

Lisa Sandmann[1,2,3,4,*,†], **Mathias Jachs**[1,5,6,†], Tammo L. Tergast[1], Lukas Hartl[5,6], Birgit Bremer[1], Martin A. Kabelitz[1], Michael Schwarz[5,6], Julius F.M. Egge[1], Lorenz Balcar[5,6], Benedikt Silvester Hofer[5,6], Christine S. Falk[4,7], Albert Friedrich Stättermayer[5,6], Markus Cornberg[1,2,3,4,8], Michael Trauner[6], Katja Deterding[1], Mattias Mandorfer[5,6], Heiner Wedemeyer[1,2,3,4], Thomas Reiberger[5,6,‡], Benjamin Maasoumy[1,3,4,‡]

JHEP Reports **2026**. vol. 8 | 1–11

Check for updates

Background & Aims: Portal hypertension (PH) drives decompensation in patients with advanced chronic liver disease. Effects of antiviral treatment with bulevirtide (BLV) and achievement of suggested treatment endpoints on PH in patients with chronic hepatitis D (CHD) are unknown.

Methods: BLV-treated CHD patients with PH were prospectively enrolled in this observational, multicenter study. Hepatic venous pressure gradient (HVPG) was measured before (BL) and after ≥12 months of BLV treatment (M12). HVPG response (≥10% decline) rates were compared between virological (VR), biochemical (BR), and combined (CR) responders *vs.* non-responders as defined in the BLV phase III studies. Associated changes in biomarkers of bacterial translocation, (dys)angiogenesis/endothelial dysfunction, and systemic inflammation (SI) were investigated.

Results: Of 34 patients receiving BL HVPG measurement, 20 patients with paired HVPG measurement and a BL clinically significant portal hypertension (CSPH) (≥10 mmHg HVPG) prevalence of 85% were included. At M12, HVPG significantly decreased in patients with CR (n = 12; 15.5 [IQR 10.5–21.8] to 12 [IQR 7.3–15.8] mmHg; p <0.001), VR (n = 14; 14.5 [IQR 10–21.3] to 12 [IQR 7.8–16.5] mmHg, p = 0.003), and BR (n = 16; 12.5 [IQR 10–20.5] to 10.5 [IQR 8–15] mmHg, p = 0.002); but not in non-responders. All patients with CSPH with CR (n = 10/10) and most of VR (83%, n = 10/12) and BR (85%, n = 11/13) achieved HVPG response, but no BLV non-responder. CSPH resolved in three of 17 (17.6%) patients. Markers of bacterial translocation (sCD163; p = 0.001), (dys)angiogenesis/endothelial dysfunction (Ang2; p = 0.001) and SI (IFNγ, IL-1RA, sCD25/IL-2Rα, CCL3/MIP-1alpha, HGF; all p <0.05) decreased in responders but not in non-responders.

Conclusions: Significant HVPG decreases accompanied by improvement of bacterial translocation, (dys)angiogenesis/endothelial dysfunction and SI are observed in patients with CHD achieving BLV response. These findings provide evidence for the validity and clinical relevance of the currently recommended on-treatment response criteria.

Clinical trials registration: This study is registered at ClinicalTrials.gov (NCT04863703).

© 2025 The Author(s). Published by Elsevier B.V. on behalf of European Association for the Study of the Liver (EASL). This is an open access article under the CC BY license (http://creativecommons.org/licenses/by/4.0/).

Introduction

Portal hypertension (PH) is a major cause of morbidity and mortality in patients with advanced chronic liver disease.[1] PH is the key driver and consequence of bacterial translocation, systemic inflammation, and immune dysfunction leading to hepatic decompensation and liver failure. In particular, patients with clinically significant portal hypertension (CSPH) are at risk of developing liver-related complications regardless of the etiology of the underlying liver disease.[2] Thus, also in rare diseases such as chronic hepatitis D (CHD), CSPH is associated with the occurrence of liver-related complications.[3] Importantly, successful treatment of the underlying liver disease is associated with improvement in PH, which translates into favorable clinical outcome.[4,5] For chronic HCV infection, antiviral treatment has been shown to significantly reduce or even normalize PH[6,7] and changes in the underlying mechanisms of PH were linked to treatment response.[8,9] With the recent approval of the entry inhibitor bulevirtide (BLV), safe and effective HDV-specific antiviral treatment for patients with

☆ Given their role as Associate Editor, Mattias Mandorfer had no involvement in the peer-review of this article and had no access to information regarding its peer-review. Full responsibility for the editorial process for this article was delegated to the Associate Editor Tobias Böttler and Editor in Chief Josep M. Llovet.

* Corresponding author. Address: Hannover Medical School, Department of Gastroenterology, Hepatology, Infectious Diseases and Endocrinology, Carl-Neuberg-Str. 1, 30625 Hannover, Germany. Tel.: +49 511 532 81672; fax: +49 511 532 4896.
E-mail address: sandmann.lisa@mh-hannover.de (L. Sandmann).
† Contributed equally and share first authorship.
‡ Contributed equally and share last authorship.
https://doi.org/10.1016/j.jhepr.2025.101643

advanced CHD and PH has become available for the first time.[10,11] Results from the phase III MYR-301 study show virological response (VR), biochemical response (BR), and combined response (CR) rates of 71%, 51%, and 45%, respectively, after 48 weeks of treatment.[12] Importantly, response rates increase with longer treatment duration.[13] However, the relevance of the applied response criteria on liver-related outcomes has not been investigated, so far.

Because of their advanced stage of liver disease at the time of diagnosis, patients with HDV and PH represent a subgroup of patients with a strong indication for antiviral treatment. Real-world studies showed that BLV is well tolerated in patients with advanced liver disease and non-invasively diagnosed CSPH.[10] Dynamics in hepatic venous pressure gradient (HVPG) are a well-established surrogate of long-term outcome in patients with liver cirrhosis.[4,14] So far, the course of PH during successful HDV suppression is insufficiently investigated. Specifically, it remains unclear if achieving response to BLV treatment leads to a reduction in HVPG or ameliorates pathophysiological mechanisms implicated in PH. In particular, the clinical relevance of achieving the currently proposed response criteria of maintenance therapy remains to be demonstrated.

The aim of this prospective, multicenter, observational study was to investigate the course of PH, assessed via paired HVPG measurements, in patients with HDV infection with cirrhosis receiving antiviral treatment with BLV.

Patients and methods

Study design and cohort

Patients with viremic CHD (anti-HDV positive, HDV RNA positive), PH (HVPG ≥ 6 mmHg), and planned start of HDV-targeted antiviral treatment with BLV were prospectively enrolled in this multicenter, observational study at Hannover Medical School (Hannover, Germany) and Medical University of Vienna (Vienna, Austria) from February 2020 to June 2023. Inclusion criteria were chronic HBV/HDV infection with detectable HDV RNA, reliable measurement of the HVPG, and planned start of antiviral treatment with BLV. Patients were included at the date of HVPG measurement before the start of BLV treatment. A second HVPG measurement was conducted after a minimum of 12 months of BLV treatment (M12). The current analysis cohort consists of all patients who underwent both HVPG measurements (Fig. 1). Clinical and laboratory data as well as liver stiffness (vibration-controlled transient elastography by FibroScan®, Echosens, Paris, France) were assessed at start of BLV treatment (baseline, BL) and M12. Decisions regarding BLV management were at the discretion of the treating physicians and not part of the study protocol of this observational study. Three patients treated at the Vienna General Hospital received add-on therapy with pegylated interferon alfa-2a (PEG-IFN) according to a local treatment policy previously described elsewhere.[15]

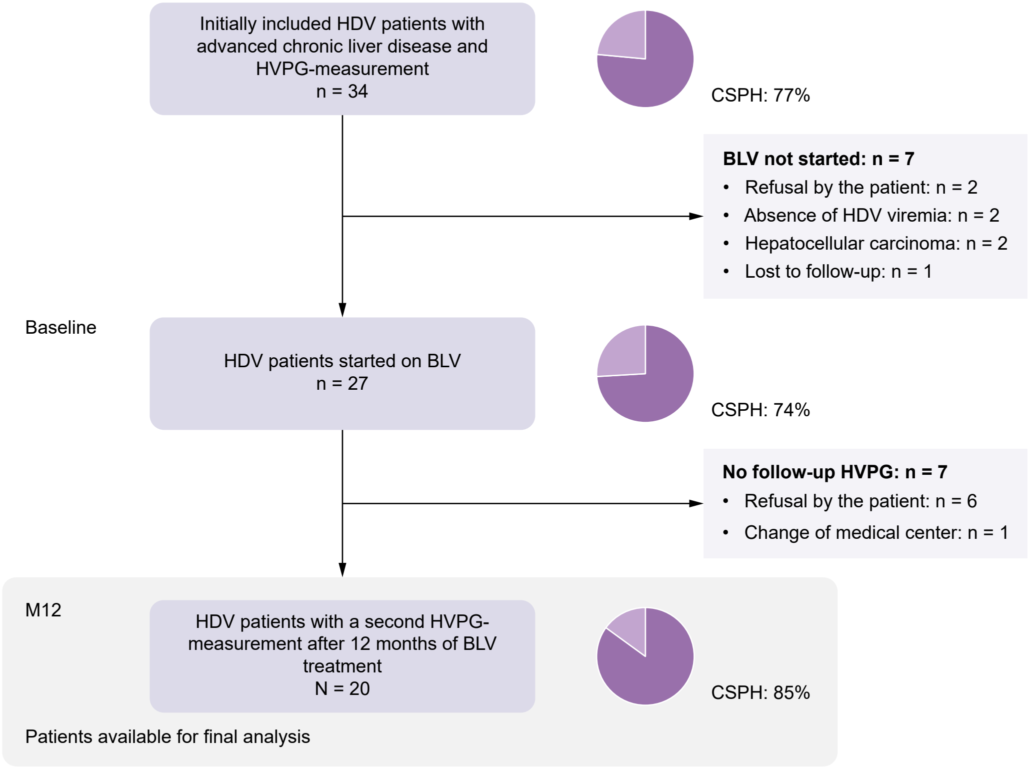

Fig. 1. Patient flow chart. BLV, bulevirtide; CSPH, clinically significant portal hypertension; HDV, hepatitis D virus; HVPG, hepatic venous pressure gradient; M12, minimum of 12 months of antiviral treatment.

Measurement of HVPG and definition of HVPG response

HVPG measurements were performed according to international recommendations.[14,16] Briefly, under local anesthesia, the right internal jugular vein was punctured and the free and wedged hepatic venous pressure was measured. The HVPG was calculated by subtracting the free hepatic venous pressure from the wedged hepatic pressure and the mean of three measurements represents the final value. In case of non-selective beta blocker treatment, the treatment was paused 5 days before HVPG measurement. Only technically reliable HVPG measurements were considered. PH was defined according to current guidelines with an HVPG of >5 mmHg defining PH and ≥10 mmHg defining CSPH.[14] A clinical meaningful change in HVPG ('HVPG response') was defined by a ≥10% decline in HVPG at the on-treatment HVPG measurement (M12).[17–19] Dynamics in HVPG were also explored in patients with subclinical PH at BL, in whom resolution of PH, that is, regression of the HVPG to normal values, is the most desirable endpoint.[4]

Evaluation of treatment response

Treatment response was assessed according to established response criteria from clinical trials[12] at the time point of the follow-up HVPG measurement (M12), with (i) VR being defined as ≥2 $\log_{10}$ HDV RNA decline or HDV RNA undetectable (TND), (ii) BR as alanine aminotransferase (ALT) normalization according to the local laboratory, and (iii) CR as the combination of VR and BR. HDV RNA was measured according to the local standards by using the RoboGene quantification assay (Robogene HDV RNA Quantification Kit 2.0, Roboscreen, Leipzig, Germany) (Hannover Medical School) or a validated in-house HDV RNA quantification assay (Medical University of Vienna). For quantitative analyses, HDV RNA levels quantified by the in-house assay were converted from cp/ml to IU/ml by applying the conversion factor of 37.[15] Importantly, local HDV RNA quantification assays remained unchanged throughout the study and virological treatment response was determined based on the original value from the local standard.

Exploratory analysis of PH-related biomarkers and systemic inflammatory markers

To investigate changes in pathophysiological mechanisms related and/or contributing to PH, markers of bacterial translocation (lipopolysaccharide-binding protein), macrophage activation (sCD163), (dys)angiogenesis/endothelial dysfunction (angiopoietin-1 [Ang1] and -2 [Ang2], vascular endothelial growth factor) and fibrogenesis (transforming growth factor beta, platelet-derived growth factor) were measured in patient serum samples before and at M12 by ELISA or as part of the 48 cytokines and chemokines Luminex-based multiplex bead assay (additional information provided in the Supplementary data). Changes in systemic inflammation (SI) were assessed by analyzing serum concentrations of 48 cytokines and chemokines by using the Luminex-based multiplex bead assay (Bio-Plex Pro™ Human Cytokine 48-plex Screening Panel, catalogue no. 12007283, BioRad Laboratories, Hercules, CA, USA) following the manufacturer's instructions. Patients with add-on PEG-IFN treatment (n = 3) were excluded for analyses of SI markers.

Statistical analysis

Categorical variables are presented as numbers and proportions and were analyzed with χ^2, Fisher's exact, or McNemar test. Continuous variables are reported as median with IQR or mean with standard deviation as indicated. Parameters were tested for normal distribution with Shapiro–Wilk test and analyzed using Mann–Whitney *U* or Wilcoxon rank statistics, or paired or unpaired *t* test as required. Values of p <0.05 were considered statistically significant. All statistical analyses were conducted using IBM SPSS Statistics (Version 28, IBM Corp., Armonk, NY, USA), GraphPad Prism version 10.2.1 for Windows (GraphPad Software, Boston, MA, USA) and R (version 4.2.0, R Foundation for Statistical Computing, Vienna, Austria)[20] with the package "ggplot2".[21]

Ethics

The study and its protocol were approved by the ethics committee of Hannover Medical School (No. 9644_BO_S_2021, NCT04863703). The Viennese patients participated in two prospective registry studies (No. 1262/2017 [NCT03267615] and 2139/2021). All patients provided written informed consent before their inclusion. The study was conducted in accordance with both the Declaration of Helsinki and Istanbul.

Results

Study cohort and treatment management

A total of 20 patients underwent paired HVPG measurement after a minimum of 12 months of BLV treatment and comprise the study cohort for this analysis (Fig. 1). At BL, patients had a median model for end-stage liver disease (MELD) of 10 points (IQR 9–12 points) and the majority was classified with Child-Pugh A cirrhosis. PH was diagnosed in all patients and 17/20 patients had CPSH (85%) (Table 1). Median time between HVPG measurement and BLV start was 59 days (IQR 20–85 days). BLV was administered at a daily dose of 2 mg and all patients received concomitant nucleos(t)ide analog treatment. PEG-IFN was added in three patients after a median treatment duration of 11 months BLV monotherapy. All of the three patients received PEG-IFN at a dose of 90 µg/week.

Response to treatment

Median MELD and Child-Pugh score did not change during treatment, whereas median levels of HDV RNA, ALT, aspartate aminotransferase (AST), and gamma-glutamyltransferase (gGT) declined significantly (Table 1). At M12, median liver stiffness measurement (LSM) values showed a substantial decline (24 kPa [IQR 13.5–28.5 kPa] *vs.* 13.5 kPa [IQR 9.0–22.7 kPa], p <0.001) and also median HVPG values significantly decreased from BL to M12 (12.5 mmHg [IQR 10–18.8 mmHg] *vs.* 10.5 mmHg [IQR 8–15.8 mmHg], p = 0.013) (Table 1). The second HVPG measurement was performed after a median of 13 months (IQR 12–17 months) of antiviral treatment. At this time point (M12), 60% (12/20), 70% (14/20), and 80% (16/20) of patients achieved CR, VR, and BR, respectively. In five of the 14 virological responders HDV RNA became undetectable. At M12, 65% (11/17) of the patients with CSPH at BL showed a clinical meaningful HVPG decline ≥10% (Table 2). Two of the three

Table 1. Baseline and on-treatment characteristics of the study cohort.

	Baseline	M12	*p* value
Patients	20		
Male	13 (65)		
Age (years)	48 (41.3–57.0)		
BMI (kg/m^2)	25.8 (22.3–29.0)		
HDV RNA (log_{10} IU/ml)	4.45 (3.35–5.57)	1.21 (0.21–2.21)	**<0.001**
HBV DNA (IU/ml)	10 (2.5–20)	0 (0–10)	**0.015**
HBsAg (log_{10} IU/ml)	4.14 (3.67–4.33)	4.0 (3.57–4.19)	**0.004**
AST (U/L)	70 (47–148)	41 (30–62)	**0.001**
ALT (U/L)	72 (62–117)	33 (23–45)	**<0.001**
Bilirubin (µmol/L)	16 (13–23)	16 (9–28)	0.896
Creatinine (µmol/L)	66 (54–82)	71 (51–85)	0.401
Albumin (g/L)	36.9 (33–40)	37.5 (34.8–42)	0.064
gGT (U/L)	55 (34–91)	34 (19–51)	**<0.001**
Platelets (G/L)	80 (39-109)	62 (39-125)	1.0
INR	1.3 (1.2-1.4)	1.3 (1.2-1.5)	0.900
Bile acids (µmol/L)	22 (6-46)	36 (20-61)	**0.001**
MELD	10 (9-12)	10 (9-13)	0.524
Child-Pugh stage			1.0
A	17 (85)	17 (85)	
B	3 (15)	3 (15)	
C	0 (0)	0 (0)	
Previous hepatic decompensation	2† (10)		
LSM (kPa)	24 (13.5-28.5)	13.5 (9.0-22.7)	**<0.001**
HVPG (mmHg)	12.5 (10-18.8)	10.5 (8-15.8)	**0.013**
PH	20 (100)	19 (95)	1.0
CSPH	17 (85)	14 (70)	0.250

Median with interquartile range for continuous parameters and numbers with percentages for categorical variables are depicted. Wilcoxon signed rank test and McNemar test were used to compare paired variables.
ALT, alanine aminotransferase; AST, aspartate aminotransferase; CSPH, clinically significant portal hypertension; HVPG, hepatic venous pressure gradient; gGT, gamma-glutamyltransferase; INR, international normalized ratio; LSM, liver stiffness measurement; MELD, model for end-stage liver disease; M12, minimum of 12 months of antiviral treatment; NA, nucleos(t)ide analog; PH, portal hypertension; TND, target not detected.
†Two patients experienced one single decompensating event (ascites: n = 1, esophageal bleeding: n = 1) 5 and 3 years before BL. Values in bold denote statistical significance.

Table 2. Treatment overview and on-treatment response rates.

**Treatment overview**	
Treatment duration (months)	13 (12–17)
Combination with PEG-IFN	3 (15)
Combined response	12 (60)
Biochemical response	16 (80)
Virological response	14 (70)
HDV RNA TND	5 (25)
HDV RNA < LLOQ	6 (30)
HDV RNA decline ≥2 log_{10}	13 (65)
HVPG response (mmHg)	-2.5 (-4.0–0)
HVPG decline ≥10%†	11 (65)

Median with interquartile range for continuous parameters and numbers with percentages for categorical variables are depicted.
HVPG, hepatic venous pressure gradient; LLOQ, lower limit of quantification; PEG-IFN, pegylated interferon alfa-2a; TND, target not detected.
†Patients with CPSH at baseline only (n = 17).

patients with subclinical PH at BL showed a numerical HVPG decline at M12, with PH fully resolving in one patient.

Significant improvement of portal hypertension in patients with treatment response

Significant improvement of PH from BL to M12 was present in the 12 patients with CR (15.5 mmHg [IQR 10.5–21.8 mmHg] to 12 mmHg [IQR 7.3–15.8 mmHg], p <0.001) whereas HVPG remained unchanged in the eight patients without CR (11 mmHg [IQR 10–14 mmHg] to 10 mmHg [8.5–17 mmHg], p = 0.5) (Fig. 2A and B). Similarly, significant HVPG improvement was only detected in the 14 patients with VR (14.5 mmHg [IQR 10–21.3 mmHg] to 12 mmHg [IQR 7.8–16.5 mmHg], p = 0.003) and in the 16 patients with BR (12.5 mmHg [IQR 10–20.5 mmHg] to 10.5 [IQR 8–15 mmHg], p = 0.002), but not in patients failing to meet these response criteria (Fig. 2C–F). Only two patients showed neither VR nor BR. In both patients, HDV RNA declined <2 log_{10} IU/ml and ALT levels increased from BL to M12. HVPG remained almost unchanged (from 11 mmHg to 10 mmHg) or increased (from 18 mmHg to 21 mmHg) from BL to M12 in these two patients.

Median HVPG change from BL to M12 was significantly higher in responders compared to non-responders (Table S1). When comparing patients with CR to patients only showing VR or BR, the median HVPG decline was significantly higher in patients with CR (-3.5 [IQR -5.75 to -2.25 mmHg] *vs.* 0.5 [IQR -0.75 to 3.0 mmHg]; p = 0.002) (Fig. S1). Importantly, median HVPG levels at BL did not differ significantly between future CR, VR, or BR responders and non-responders (Table S1). Among the patients with CSPH at BL, HVPG response was achieved by all patients who showed CR (n = 10/10), and most of virological (83%, n = 10/12) and biochemical responders (85%, n = 11/13). The proportion of CSPH patients showing a clinical meaningful HVPG response was significantly higher in patients with CR (100%, n = 10/10) compared with patients showing VR or BR only (20%, n = 1/5; p = 0.004). Meanwhile, none of the CSPH patients without any treatment response (CR/VR/BR) achieved HVPG response at M12. CSPH resolved in 18% (3/17) of patients with CSPH at BL.

Clinical outcome during follow-up

Clinical follow-up data was available for all patients with a median follow-up time of 22 (IQR 14.1–32.8) months after M12. During follow-up, only one patient developed a decompensating event (ascites) after M12. This patient did not show any treatment response (neither VR nor BR) and HVPG was increasing from BL to M12. The patient developed further decompensation and received liver transplantation 9 months after the initial decompensating event. None of the other patients showed any decompensating event during follow-up. Hepatocellular carcinoma was diagnosed in four patients after a median treatment duration of BLV of 18.8 (IQR 16–24.9) months.

Concordance of HVPG measurement and non-invasive test assessment before and during treatment with BLV

Non-invasive tests (NITs) can be applied to detect or exclude CSPH in patients with CHD.[3] Whether on-treatment NIT assessment reliably detects PH amelioration during antiviral treatment of CHD is unclear. From BL to M12, median LSM values declined significantly in patients with treatment response (Table S2). They remained unchanged in patients without VR or BR, but also decreased in patients without CR (20.8 kPa [IQR 11–26 kPa] to 14.4 kPa [IQR 7.3–25 kPa], p = 0.039). The two patients without any treatment response showed divergent LSM, with one showing an increase and the other a decline. No significant differences were detected when comparing LSM results of responders and non-responders at

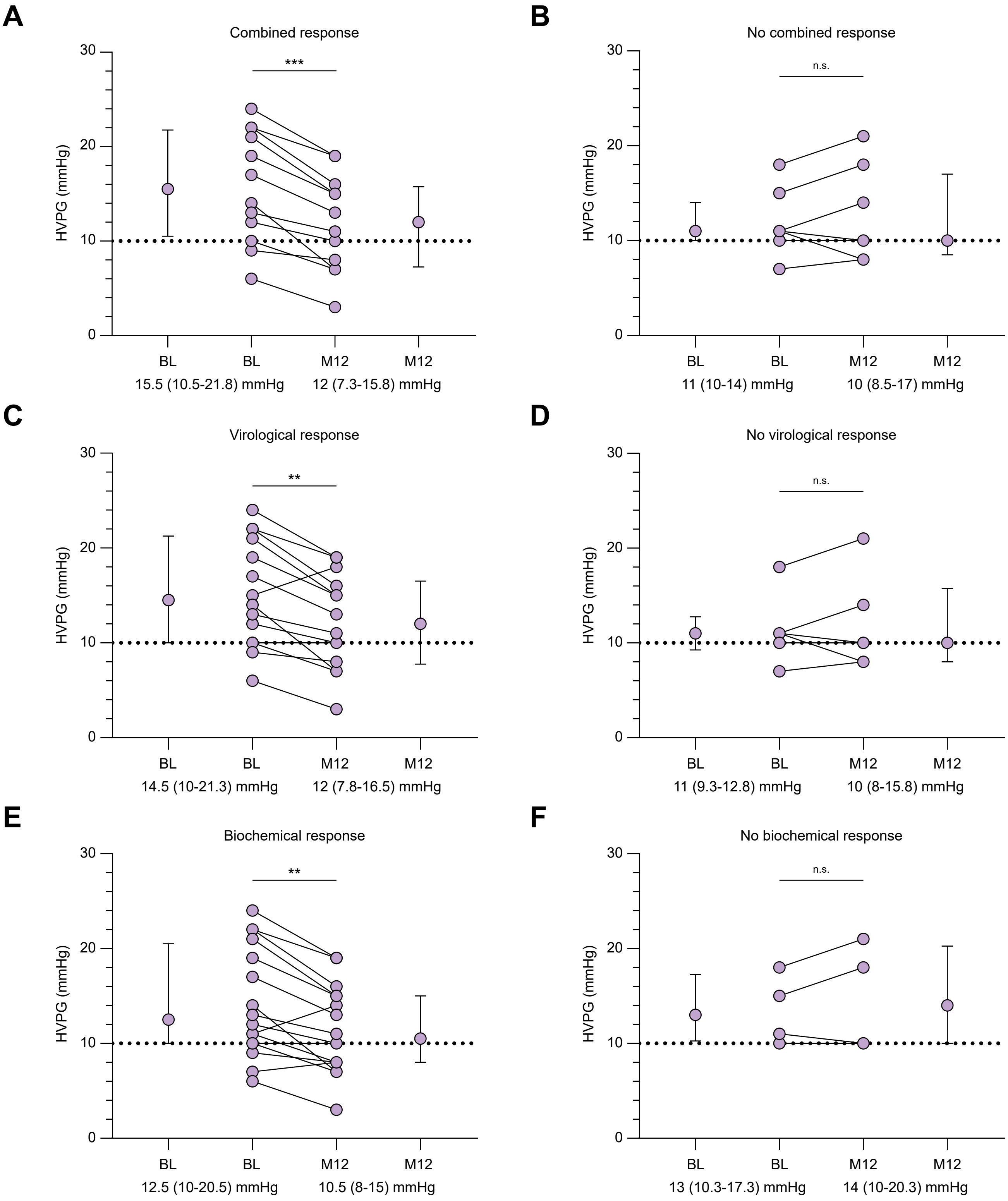

Fig. 2. Course of portal hypertension according to bulevirtide response status. Comparison of HVPG at baseline and M12 of patients with (A) or without (B) combined response, with (C) or without (D) virological response, and with (E) or without (F) biochemical response. Medians with interquartile range and individual values are depicted. Wilcoxon signed rank test was used to compare baseline and M12 results. ***p* <0.01; ****p* <0.001; n.s., not significant.

BL and M12. However, the difference in median LSM between BR and non-responders at M12 almost reached statistical significance (Table S3).

When applying the widely used non-invasive Baveno VII criteria for CSPH ruling-in (LSM ≥25 kPa) and ruling-out (LSM ≤15 kPa and platelet count ≥150 G/L)[14] to the cohort, the specificity and positive predictive values for the 'ruled-in' of CSPH were 100% at BL and M12. Similarly, a reliable exclusion based on the 'ruled-out' criteria was observed at BL and M12 (Table S4). However, the proportion of patients in the gray zone increased from 55% at BL to 75% at M12. None of the three patients with CPSH resolution at M12 would have been classifiable by NIT assessment, as all were in the gray zone.

Treatment-induced changes of PH-related biomarkers

To investigate changes in pathophysiological mechanism contributing to PH, biomarkers reflecting bacterial translocation, macrophage activation, (dys)angiogenesis, endothelial dysfunction, and fibrogenesis were analyzed. With ongoing antiviral treatment, median levels of sCD163, a marker reflecting macrophage activation, declined significantly. Interestingly, this effect was only observed in patients with treatment response and irrespective of the classification of response (Fig. 3A). Furthermore, median levels of the (dys) angiogenesis and endothelial dysfunction marker Ang2 declined significantly from BL to M12. This decline was only observed in the subgroups of patients with treatment response and not in those without (Fig. 3B). No significant differences were observed when comparing BL and M12 levels of LBP, TGFb, PDGF, VEGF, Ang1, or the Ang2/Ang1 ratio (Table S5). Median cytokine levels at BL did not differ between future combined, virological, or biochemical responders and non-responders (Table S6). Similar results were obtained when stratifying according to clinical meaningful HVPG response (Table S7).

Changes of systemic inflammation in patients with treatment response

No significant differences were detected for levels of C-reactive protein or white blood cell count when comparing BL and M12 results of treatment responders and non-responders (Table S8). However, when comparing the comprehensive panel of SI markers during antiviral treatment, CR and non-responders showed different patterns of SI changes (Fig. 4A and B). In patients with CR, significant declines of interleukin-1 receptor antagonist (IL-1RA), interleukin-2 receptor alpha (sCD25), interferon gamma (IFNγ), hepatocyte growth factor (HGF), and macrophage inflammatory protein-1 alpha (CCL3/MIP-1a) were detected from BL to M12, while no significant differences were present in patients without CR. Comparable patterns of SI changes were present in patients with VR and BR (Fig. 4C and E). By contrast, patients without treatment

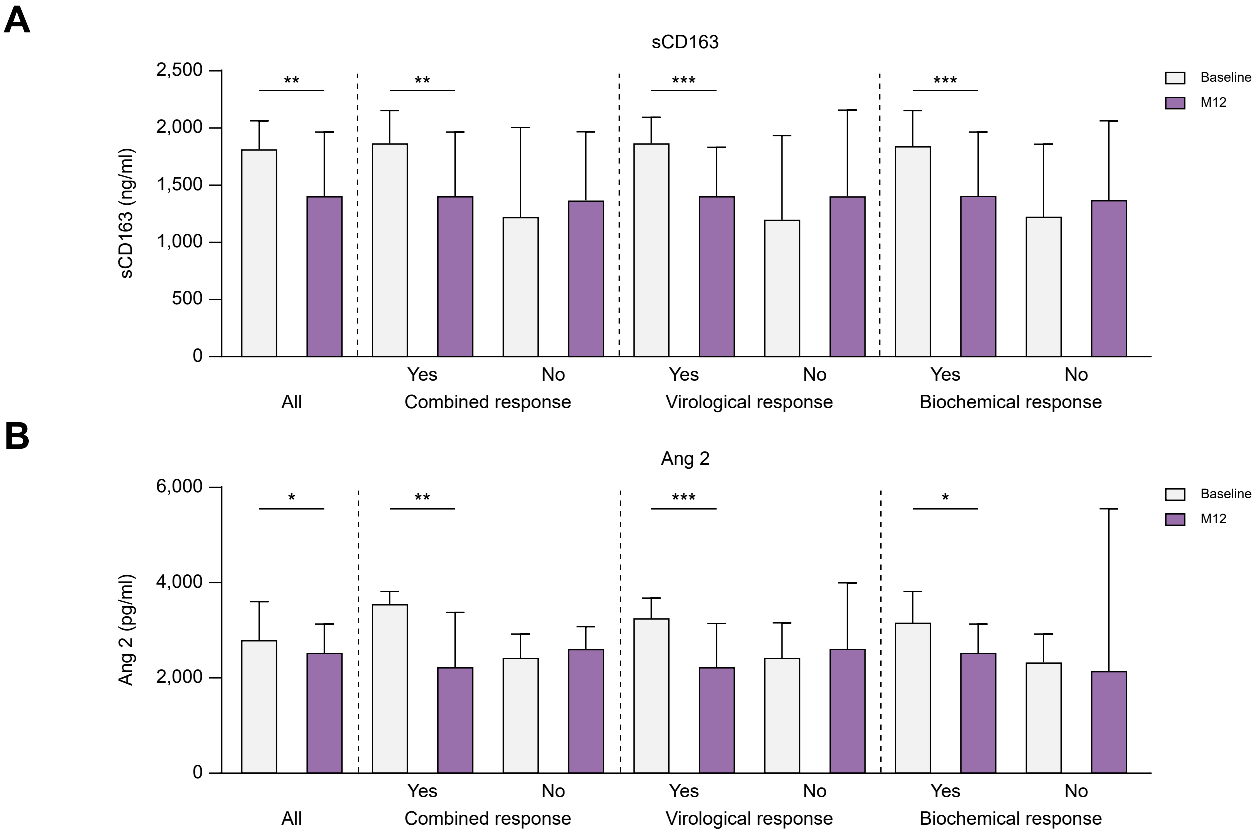

Fig. 3. Comparison of PH-related biomarkers reflecting macrophage activation and (dys)angiogenesis. Comparison of baseline and M12 levels of sCD163 (A) and Ang2 (B) of all patients, and grouped according to treatment response. Medians with interquartile range are depicted. Wilcoxon signed rank test was used to compare baseline and M12 parameters. *p <0.05; **p <0.01; ***p <0.001. Ang2, angiopoietin-2; M12, minimum of 12 months of antiviral treatment; sCD163, soluble cluster of differentiation 163.

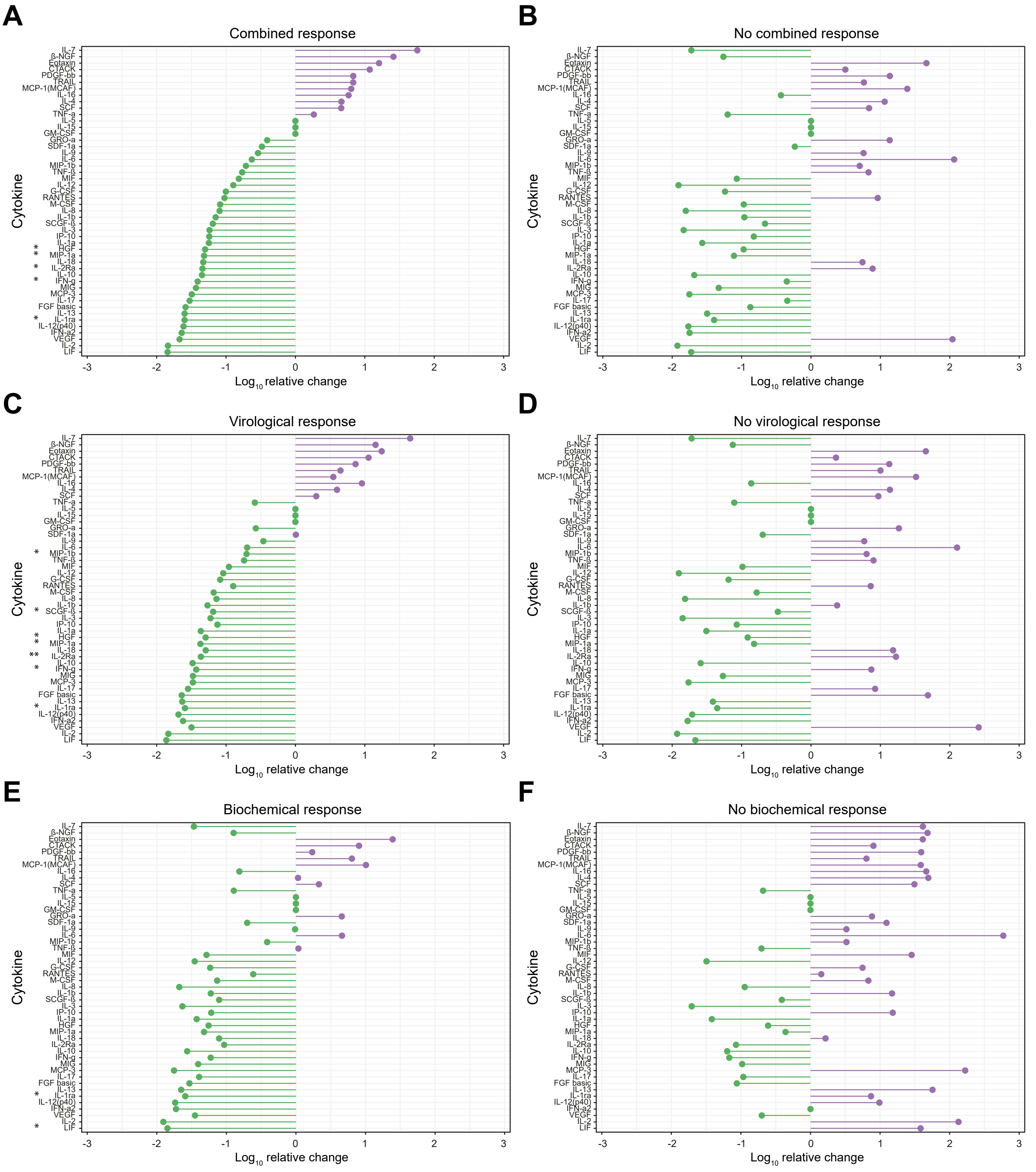

Fig. 4. Changes in circulating systemic inflammation profile according to bulevirtide response status. Relative mean change of systemic inflammation markers is depicted for patients with (A) and without (B) combined response, with (C) and without (D) virological response, and with (E) and without (F) biochemical response. Wilcoxon signed rank test was used to compare baseline and M12 results. **p* <0.05; ***p* <0.01. BL, baseline; M12, minimum of 12 months of antiviral treatment.

response showed a strong numerical increase of IL-6 levels from BL to M12. This increase was highest in patients without BR.

Discussion

In this prospective, observational study of CHD patients with PH treated with BLV for a minimum of 12 months, response to treatment according to endpoints applied in clinical studies on BLV treatment was associated with clinically meaningful HVPG decreases in all or most of the patients who achieved CR or VR/BR, respectively. Meanwhile, none of the non-responders achieved HVPG response.

PH is the driving mechanism of clinical deterioration in patients with advanced chronic liver disease. Particularly, patients with CSPH are at risk of developing decompensating events thereby leading to increased morbidity and mortality.[2] Also, in patients with CHD, CSPH is associated with the occurrence of decompensating events and worse outcome.[3] With the availability of BLV the treatment landscape of CHD has changed significantly.[22,23] The treatment is well tolerated and response rates increase with longer treatment duration even in patients with CSPH in whom previous treatment options, that is, PEG-IFN, were often poorly tolerated.[13] Still, treatment duration is unclear and treatment response is evaluated based on the surrogate endpoints HDV RNA response (VR), ALT normalization (BR), or their combination (CR).[24] Notably, particularly the decrease in HDV RNA by ≥2 $\log_{10}$ units was arbitrarily defined owing to *post-hoc* analyses of the first landmark study evaluating interferon treatment in CHD.[25,26] Interestingly, interferon treatment *per se* has previously been associated with clinical benefit irrespective of treatment response.[27] Meanwhile, long-term follow-up studies investigating hard clinical endpoints such as improvement of patient survival by the achievement of response to treatment are lacking. HVPG response is a well-established surrogate for clinical benefit and is recommended for clinical studies on PH according to international consensus.[14] Intriguingly, all patients who achieved CR in our cohort also achieved HVPG response. Our findings corroborate the previously demonstrated findings for chronic HCV infection, where several studies investigated the effect of antiviral treatment on PH either evaluated by HVPG measurement[4,7,28] or NITs.[29,30] Interestingly, the achievement of HVPG response protected patients from future hepatic decompensation,[4] which is also demonstrated by the clinical follow-up data of our cohort.

Importantly, recent data have shown that also in CHD, the absence of CSPH identifies a subgroup of patients with no or only very low risk of developing decompensating events.[3] Therefore, the high rate of clinical meaningful HVPG decline in patients responding to antiviral treatment, as shown in the present study, strengthens the clinical relevance of the suggested on-treatment response criteria. Meanwhile, we observed ubiquitous decreases in LSM, the most relevant CSPH-NIT, in all patients, including those without CR. Notably, early LSM decline during antiviral treatment is most likely linked to the regression of hepatic inflammation, for which ALT decline or ALT normalization is a surrogate marker.[31,32] Patients without CR can still show BR, which explains the previously mentioned findings. The extent to which and at what point during antiviral treatment the decline in LSM actually reflects regression of fibrosis would need to be investigated in future studies. Along this, future studies comprising larger cohorts may focus on the diagnostic value of LSM in predicting events during or following BLV treatment in CHD, similar to the studies conducted for chronic HCV infection and antiviral treatment.[4,6,28,29]

Little is known about the impact of antiviral treatment in CHD on the pathophysiological mechanisms of PH. In our study, markers of macrophage activation (sCD163) and (dys) angiogenesis (Ang2) declined significantly during treatment in patients with treatment response. Both parameters have been investigated in the context of chronic HCV infection and antiviral treatment with declining levels being associated with treatment response.[8,33] Furthermore, levels of sCD163 positively correlated with the severity of PH in patients with cirrhosis.[34,35] Circulating Ang2 levels decreased in patients achieving HCV cure and were linked to an improvement in PH in patients with CSPH before antiviral treatment.[8] Accordingly, higher Ang2 levels after antiviral treatment were associated with post-treatment hepatic decompensation. Our results are consistent with published data on chronic HCV and HBV infection. However, it is not possible at present to distinguish whether the changes are due to the resolution of hepatic inflammation as a result of antiviral treatment or to a regression of fibrosis. Interestingly, analyses of SI biomarkers revealed different patterns of change in treatment responders and non-responders. By reducing the viral load, antiviral treatment leads to pathogen reduction and regression of liver inflammation, which could also be reflected in changes of SI biomarker. In addition, improvement of PH could also lead to a reduction of systemic inflammation. PH is associated with systemic inflammation,[36] and effective treatment of PH has been shown to reduce systemic inflammation.[35] In our cohort, the most striking differences appear to be present in declining levels of IL-1RA, sCD25/interleukin-2 receptor alpha, IFNγ, HGF, and CCL3/MIP-1a in responders and increasing levels of IL-6 in non-responders. Data on dynamics in SI during antiviral treatment in CHD are scarce. Three studies investigated selected cytokines during PEG-IFN treatment[37,38] of which only one included off-treatment data.[39] In the latter, mean levels of IFNγ did not change significantly between BL and 6 months off-treatment in patients with VR. However, comparability to our study is questionable, as treatment regimen and response criteria differed. Other markers have been investigated in the context of chronic HCV infection and antiviral treatment. Higher levels of IL-1RA were present in patients with ongoing HCV infection, while declining levels were observed after HCV cure.[40,41] Similarly, declining levels of CCL3/MIP-1a were observed in patients with HCV receiving antiviral treatment,[42,43] whereas higher levels of HGF were detected in patients with more advanced liver disease irrespective of the etiology of liver disease.[44] Overall, relative decrease of SI was present in patients with treatment response in our cohort. By contrast, non-responders showed a relative increase in IL-6, a pro-inflammatory cytokine that has been shown to be elevated in patients with advanced liver disease and thus serves as a predictor of decompensating events.[45]

Our study has limitations. First, we could only include a relatively small number of patients. It is possible that a larger cohort would have led to different results in the analysis of exploratory endpoints such as LSM or markers of systemic inflammation. However, CHD is an orphan disease and HVPG

measurements are only performed in specialized centers. Importantly, the observed results regarding the HVPG response were striking, clearly linking the achievement of surrogate treatment endpoints to HVPG response even in a limited number of patients. Second, the treatment decision was based on the treating physician's decision and was not part of the study protocol of this observational study. Therefore, three patients received add-on treatment with PEG-IFN after a median of 11 months of BLV monotherapy. As the main objective of this study was to investigate the impact of treatment response on HVPG regardless of treatment regimen, we do not see any relevant impact on the main findings of this study. Two of the three patients receiving add-on PEG-IFN showed VR, BR, and CR whereas one patient was classified as a non-responder (virological and biochemical non-response). Importantly, patients receiving add-on PEG-IFN treatment were excluded from analyses of SI markers, as a considerable confounding effect of PEG-IFN is expected. Additionally, BR and CR rates in our study were higher than those reported in the phase III clinical study. These differences are most likely explained by the differences between the cohorts. Our study exclusively included patients with cirrhosis and PH, whereas only 47% of the patients in the clinical study had liver cirrhosis. Additionally, median ALT levels at baseline were lower in our cohort compared with the phase III clinical study, which can be explained by the higher rates of advanced liver disease. Overall lower ALT levels at baseline are likely to result in higher rates of normalized ALT levels at follow-up, which explains the higher rate of BR and CR in our study cohort. When comparing our results with those of other real-life cohorts including patients with advanced liver disease, the VR and BR rates were comparable.[46]

In conclusion, this prospective observational study demonstrates that response to antiviral treatment with BLV in patients with CHD and PH is associated with a clinically meaningful decrease in HVPG and improvement of biomarkers reflecting liver disease activity and progression. The findings of our study provide for the first time strong evidence for the validity and clinical relevance of the currently suggested response criteria for antiviral therapy in advanced CHD, as their achievement is linked to a HVPG response as a key surrogate for clinical benefit in regard to favorable long-term outcomes.

Affiliations

[1]*Department of Gastroenterology, Hepatology, Infectious Diseases and Endocrinology, Hannover Medical School, Hannover, Germany;* [2]*D-SOLVE Consortium, An EU Horizon Europe-funded Project (no. 101057917), Hannover, Germany;* [3]*Excellence Cluster RESIST, Hannover Medical School, Hannover, Germany;* [4]*German Center for Infection Research (DZIF), Hannover/Braunschweig, Germany;* [5]*Vienna Hepatic Hemodynamic Lab, Division of Gastroenterology and Hepatology, Department of Medicine III, Medical University of Vienna, Vienna, Austria;* [6]*Division of Gastroenterology and Hepatology, Department of Medicine III, Medical University of Vienna, Vienna, Austria;* [7]*Institute of Transplant Immunology, Hannover Medical School, Hannover, Germany;* [8]*Center of Individualised Infection Medicine, A Joint Venture Between Helmholtz-Centre for Infection Research and Hannover Medical School, Hannover, Germany*

Abbreviations

ALT, alanine aminotransferase; Ang1, angiopoietin-1; Ang2, angiopoietin-2; AST, aspartate aminotransferase; BL, baseline; BLV, bulevirtide; BR, biochemical response; CHD, chronic hepatitis delta; CR, combined response; CSPH, clinically significant portal hypertension; gGT, gamma-glutamyltransferase; HGF, hepatocyte growth factor; HVPG, hepatic venous pressure gradient; IFNγ, interferon gamma; IL-1RA, interleukin-1 receptor antagonist; INR, international normalized ratio; LSM, liver stiffness measurement; M12, treatment month 12; MELD, model for end-stage liver disease; MIP-1a, macrophage inflammatory protein-1 alpha; NITs, non-invasive tests; PEG-IFN, pegylated interferon alfa-2a; PH, portal hypertension; SI, systemic inflammation; TND, target not detected; VR, virological response.

Financial support

LS was partially funded by the Advanced Clinician Scientist program of the German Center for Infection Research (DZIF). The study was partially funded by HiLF I of Hannover Medical School.

Conflicts of interest

The authors declare no conflict of interest regarding this study. Outside the submitted work, the authors declare the following potential conflicts of interest: LS reports lecture honoraria and personal fees from Falk Pharma e.V., Gilead and Roche, and travel support from AbbVie and Gilead. MJ served as a speaker and consultant for Gilead. TLT served as speaker for Falk Pharma GmbH. MSch received travel support from MSD, Sandoz, BMS, AbbVie, and Gilead; received speaking honoraria from BMS and Gilead; and served as a consultant for Gilead. BSH reports travel support by Ipsen. MC reports personal fees from AbbVie, Falk Foundation, Gilead, Janssen-Cilag, GSK, MSD, Spring Bank, and SOBI. MT served as a speaker and/or consultant and/or advisory board member for Agomab, Albireo, BiomX, Falk, Boehringer Ingelheim, Bristol-Myers Squibb, Chemomab, Falk, Genfit, Gilead, Intercept, Janssen, MSD, Madrigal, Novartis, Phenex, Pliant, Regulus, and Shire, and received travel support from AbbVie, Falk, Gilead, and Intercept, as well as grants/research support from Albireo, Alnylam, Cymabay, Falk, Gilead, Intercept, MSD, Takeda, and UltraGenyx. He is also co-inventor of patents on the medical use of 24-norursodeoxycholic acid filed by the Medical Universities of Graz and Vienna. KD received lecture and personal fees from Gilead, Falk Pharma e.V., AbbVie, MSD/Merck, and Alnylam. MM served as a speaker and/or consultant and/or advisory board member for AbbVie, AstraZeneca, Echosens, Eli Lilly, Falk, Gilead, Ipsen, Takeda, and W.L. Gore & Associates and received travel support from AbbVie and Gilead as well as grants/research support from Echosens. HW has received fees for lectures and/or consultations from AbbVie, Aligos, Altimmune, Biotest, BMS, BTG, Dicerna, Enanta, Gilead, Janssen, Merck/MSD, MYR GmbH, Roche, and Vir Biotechnology. TR served as a speaker and/or consultant and/or advisory board member for AbbVie, Bayer, Boehringer Ingelheim, Gilead, Intercept, MSD, Siemens, and W.L. Gore & Associates and received grants/research support from AbbVie, Boehringer Ingelheim, Gilead, Intercept, MSD, Myr Pharmaceuticals, Pliant, Philips, Siemens, and W.L. Gore & Associates as well as travel support from AbbVie, Boehringer Ingelheim, Gilead, and Roche. BM served as a speaker and/or advisory board member for AbbVie, AstraZeneca, Fujirebio, Gilead, Luvos, MSD, Norgine, Roche, W.L. Gore & Associates and received research support from Altona, EWIMED, Fujirebio, and Roche. The remaining authors declare no conflicts of interest that pertain to this work.

Please refer to the accompanying ICMJE disclosure forms for further details.

Authors' contributions

Conception and design of the study: LS, MJ, TLT, TR, BM. Funding acquisition: LS, BM. Acquisition of data: LS, MJ, M S, LB, BSH, AFS, JFME, BB, CSF, KD, MM, BM. Data analysis: LS, MJ, MAK. Interpretation of data: LS, MJ, HW, TR, BM. Drafting of the manuscript: LS, MJ. Reviewing and editing: all authors. Final approval of the manuscript: all authors.

Data availability

Data are available upon reasonable request.

Acknowledgements

We thank Julia Schneider and Janet Cornberg for their assistance in patient management and clinical data collection.

Supplementary data

Supplementary data to this article can be found online at https://doi.org/10.1016/j.jhepr.2025.101643.

References

Author names in bold designate shared co-first authorship

[1] D'Amico G, Garcia-Tsao G, Pagliaro L. Natural history and prognostic indicators of survival in cirrhosis: a systematic review of 118 studies. J Hepatol 2006;44:217–231.

[2] Ripoll C, Groszmann R, Garcia-Tsao G, et al. Hepatic venous pressure gradient predicts clinical decompensation in patients with compensated cirrhosis. Gastroenterology 2007;133:481–488.

[3] **Jachs M**, **Sandmann L**, Hartl L, et al. Validation of Baveno VII criteria and other non-invasive diagnostic algorithms for clinically significant portal hypertension in hepatitis delta. J Hepatol 2024;81:248–257.

[4] Mandorfer M, Kozbial K, Schwabl P, et al. Changes in hepatic venous pressure gradient predict hepatic decompensation in patients who achieved sustained virologic response to interferon-free therapy. Hepatology 2020;71:1023–1036.

[5] Manolakopoulos S, Triantos C, Theodoropoulos J, et al. Antiviral therapy reduces portal pressure in patients with cirrhosis due to HBeAg-negative chronic hepatitis B and significant portal hypertension. J Hepatol 2009;51:468–474.

[6] Mandorfer M, Kozbial K, Schwabl P, et al. Sustained virologic response to interferon-free therapies ameliorates HCV-induced portal hypertension. J Hepatol 2016;65:692–699.

[7] Lens S, Baiges A, Alvarado-Tapias E, et al. Clinical outcome and hemodynamic changes following HCV eradication with oral antiviral therapy in patients with clinically significant portal hypertension. J Hepatol 2020;73:1415–1424.

[8] Bauer D, Kozbial K, Schwabl P, et al. Angiopoietin 2 levels decrease after HCV-cure and reflect the evolution of portal hypertension. Dig Liver Dis 2022;54:1222–1229.

[9] Schwabl P, Mandorfer M, Steiner S, et al. Interferon-free regimens improve portal hypertension and histological necroinflammation in HIV/HCV patients with advanced liver disease. Aliment Pharmacol Ther 2017;45:139–149.

[10] Degasperi E, Anolli MP, Uceda Renteria SC, et al. Bulevirtide monotherapy for 48 weeks in patients with HDV-related compensated cirrhosis and clinically significant portal hypertension. J Hepatol 2022;77:1525–1531.

[11] Lampertico P, Roulot D, Wedemeyer H. Bulevirtide with or without pegIFNalpha for patients with compensated chronic hepatitis delta: from clinical trials to real-world studies. J Hepatol 2022;77:1422–1430.

[12] Wedemeyer H, Aleman S, Brunetto MR, et al. A phase 3, randomized trial of bulevirtide in chronic hepatitis D. N Engl J Med 2023;389:22–32.

[13] Wedemeyer H, Aleman S, Brunetto M, et al. Bulevirtide monotherapy in patients with chronic HDV: efficacy and safety results through week 96 from a phase III randomized trial. J Hepatol 2024;81:621–629.

[14] de Franchis R, Bosch J, Garcia-Tsao G, et al. Baveno VII – renewing consensus in portal hypertension. J Hepatol 2022;76:959–974.

[15] Jachs M, Schwarz C, Panzer M, et al. Response-guided long-term treatment of chronic hepatitis D patients with bulevirtide-results of a "real world" study. Aliment Pharmacol Ther 2022;56:144–154.

[16] Bettinger D, Berzigotti A, Mandorfer M, et al. Transjugular diagnostic procedures in hepatology: indications, techniques and interpretation. JHEP Rep 2025;7:101437.

[17] de Franchis R, Baveno VI. Faculty Expanding consensus in portal hypertension: report of the Baveno VI Consensus Workshop: stratifying risk and individualizing care for portal hypertension. J Hepatol 2015;63:743–752.

[18] Groszmann RJ, Garcia-Tsao G, Bosch J, et al. Beta-blockers to prevent gastroesophageal varices in patients with cirrhosis. N Engl J Med 2005;353:2254–2261.

[19] Villanueva C, Albillos A, Genesca J, et al. Beta blockers to prevent decompensation of cirrhosis in patients with clinically significant portal hypertension (PREDESCI): a randomised, double-blind, placebo-controlled, multicentre trial. Lancet 2019;393:1597–1608.

[20] R Core Team. R: a language and environment for statistical computing. Vienna, Austria: R Foundation for Statistical Computing; 2018. https://www.R-project.org.

[21] Wickham H. ggplot2 – elegant graphics for data analysis. Springer; 2016.

[22] European Association for the Study of the Liver. EASL Clinical Practice Guidelines on hepatitis delta virus. J Hepatol 2023;79:433–460.

[23] Sandmann L, Berg T, Deterding K, et al. Antiviral therapy of chronic hepatitis D virus infection – addendum to the S3 guideline "Prophylaxis, diagnosis and therapy of hepatitis B virus infection" of the German society for gastroenterology, digestive and metabolic diseases (DGVS). Z Gastroenterol 2023;61:e715–e732.

[24] Ghany MG, Buti M, Lampertico P, et al. Guidance on treatment endpoints and study design for clinical trials aiming to achieve cure in chronic hepatitis B and D: report from the 2022 AASLD-EASL HBV-HDV Treatment Endpoints Conference. Hepatology 2023;78:1654–1673.

[25] Farci P, Roskams T, Chessa L, et al. Long-term benefit of interferon alpha therapy of chronic hepatitis D: regression of advanced hepatic fibrosis. Gastroenterology 2004;126:1740–1749.

[26] Sandmann L, Wedemeyer H. Interferon-based treatment of chronic hepatitis D. Liver Int 2023;43(Suppl 1):69–79.

[27] Wranke A, Serrano BC, Heidrich B, et al. Antiviral treatment and liver-related complications in hepatitis delta. Hepatology 2017;65:414–425.

[28] Lens S, Alvarado-Tapias E, Marino Z, et al. Effects of all-oral anti-viral therapy on HVPG and systemic hemodynamics in patients with hepatitis C virus-associated cirrhosis. Gastroenterology 2017;153:1273–12783.e1.

[29] Semmler G, Binter T, Kozbial K, et al. Noninvasive risk stratification after HCV eradication in patients with advanced chronic liver disease. Hepatology 2021;73:1275–1289.

[30] Semmler G, Alonso Lopez S, Pons M, et al. Long-term outcome and risk stratification in compensated advanced chronic liver disease after HCV-cure. Hepatology 2025;81:609–624.

[31] **Pietsch V**, **Deterding K**, Attia D, et al. Long-term changes in liver elasticity in hepatitis C virus-infected patients with sustained virologic response after treatment with direct-acting antivirals. United Eur Gastroenterol J 2018;6:1188–1198.

[32] Bachofner JA, Valli PV, Kroger A, et al. Direct antiviral agent treatment of chronic hepatitis C results in rapid regression of transient elastography and fibrosis markers fibrosis-4 score and aspartate aminotransferase-platelet ratio index. Liver Int 2017;37:369–376.

[33] Lund Laursen T, Brockner Siggard C, Kazankov K, et al. Rapid and persistent decline in soluble CD163 with successful direct-acting antiviral therapy and associations with chronic hepatitis C histology. Scand J Gastroenterol 2018;53:986–993.

[34] Gronbaek H, Sandahl TD, Mortensen C, et al. Soluble CD163, a marker of Kupffer cell activation, is related to portal hypertension in patients with liver cirrhosis. Aliment Pharmacol Ther 2012;36:173–180.

[35] Tiede A, Stockhoff L, Liu Z, et al. Insertion of a transjugular intrahepatic portosystemic shunt leads to sustained reversal of systemic inflammation in patients with decompensated liver cirrhosis. Clin Mol Hepatol 2025;31:240–255.

[36] Albillos A, Martin-Mateos R, Van der Merwe S, et al. Cirrhosis-associated immune dysfunction. Nat Rev Gastroenterol Hepatol 2022;19:112–134.

[37] Lutterkort GL, Wranke A, Hengst J, et al. Viral dominance patterns in chronic hepatitis delta determine early response to interferon alpha therapy. J Viral Hepat 2018;25:1384–1394.

[38] Anastasiou OE, Yurdaydin C, Maasoumy B, et al. A transient early HBV-DNA increase during PEG-IFNalpha therapy of hepatitis D indicates loss of infected cells and is associated with HDV-RNA and HBsAg reduction. J Viral Hepat 2021;28:410–419.

[39] Grabowski J, Yurdaydin C, Zachou K, et al. Hepatitis D virus-specific cytokine responses in patients with chronic hepatitis delta before and during interferon alfa-treatment. Liver Int 2011;31:1395–1405.

[40] Medrano LM, Berenguer J, Salguero S, et al. Successful HCV therapy reduces liver disease severity and inflammation biomarkers in HIV/HCV-coinfected patients with advanced cirrhosis: a cohort study. Front Med (Lausanne) 2021;8:615342.

[41] Lara-Aguilar V, Crespo-Bermejo C, Llamas-Adan M, et al. HCV spontaneous clearers showed low senescence profile in people living with HIV under long ART. J Med Virol 2023;95:e28955.

[42] Jimenez-Sousa MA, Almansa R, de la Fuente C, et al. Increased Th1, Th17 and pro-fibrotic responses in hepatitis C-infected patients are down-regulated after 12 weeks of treatment with pegylated interferon plus ribavirin. Eur Cytokine Netw 2010;21:84–91.

[43] Ribeiro IG, Coelho-Dos-Reis JGA, Fradico JRB, et al. Remodeling of immunological biomarkers in patients with chronic hepatitis C treated with direct-acting antiviral therapy. Antivir Res 2021;190:105073.

[44] Latorre R, Vaquero J, Rincon D, et al. Determinants of platelet count are different in patients with compensated and decompensated cirrhosis. Liver Int 2016;36:232–239.

[45] Costa D, Simbrunner B, Jachs M, et al. Systemic inflammation increases across distinct stages of advanced chronic liver disease and correlates with decompensation and mortality. J Hepatol 2021;74:819–828.

[46] Dietz-Fricke C, Degasperi E, Jachs M, et al. Safety and efficacy of off-label bulevirtide monotherapy in patients with HDV with decompensated Child-B cirrhosis –a real-world case series. Hepatology 2024;80:664–673.

Keywords: HDV; CSPH; HVPG; Viral hepatitis.
Received 20 May 2025; received in revised form 6 October 2025; accepted 13 October 2025; Available online 17 October 2025

Journal of Hepatology, Volume *8*

Supplemental information

Treatment response to bulevirtide is linked to amelioration of portal hypertension in patients with chronic hepatitis D

Lisa Sandmann, Mathias Jachs, Tammo L. Tergast, Lukas Hartl, Birgit Bremer, Martin A. Kabelitz, Michael Schwarz, Julius F.M. Egge, Lorenz Balcar, Benedikt Silvester Hofer, Christine S. Falk, Albert Friedrich Stättermayer, Markus Cornberg, Michael Trauner, Katja Deterding, Mattias Mandorfer, Heiner Wedemeyer, Thomas Reiberger, and Benjamin Maasoumy

Treatment response to bulevirtide is linked to amelioration of portal hypertension in patients with chronic hepatitis D☆

Lisa Sandmann, Mathias Jachs, Tammo L. Tergast, Lukas Hartl, Birgit Bremer, Martin A. Kabelitz, Michael Schwarz, Julius F.M. Egge, Lorenz Balcar, Benedikt Silvester Hofer, Christine S. Falk, Albert Friedrich Stättermayer, Markus Cornberg, Michael Trauner, Katja Deterding, Mattias Mandorfer, Heiner Wedemeyer, Thomas Reiberger, Benjamin Maasoumy

Table of contents

Supplementary methods 2
Fig. S1 3
Table S1. 4
Table S2. 5
Table S3. 6
Table S4. 7
Table S5. 8
Table S6. 12
Table S7. 15
Table S8. 17

Supplementary methods

Exploratory analysis of PH-related biomarkers and systemic inflammatory markers

The following ELISAs were used according to the manufacturers' instructions: LBP (ab213805, Abcam, Cambridge, UK), CD163 (Quantikine® ELISA Human CD163 Immunoassay, R&D Systems, Catalog Number DC 1630), Ang1 (Quantikine® ELISA Human Angiopoietin-1 Immunoassay, R&D Systems, Catalog Number DANG10), Ang2 (Quantikine® ELISA Human Angiopoietin-2 Immunoassay, R&D Systems, Catalog Number DANG20), and TGFb (Quantikine® ELISA Human TGF-β1 Immunoassay, R&D Systems, Catalog Number DB100C).

Supplementary figures

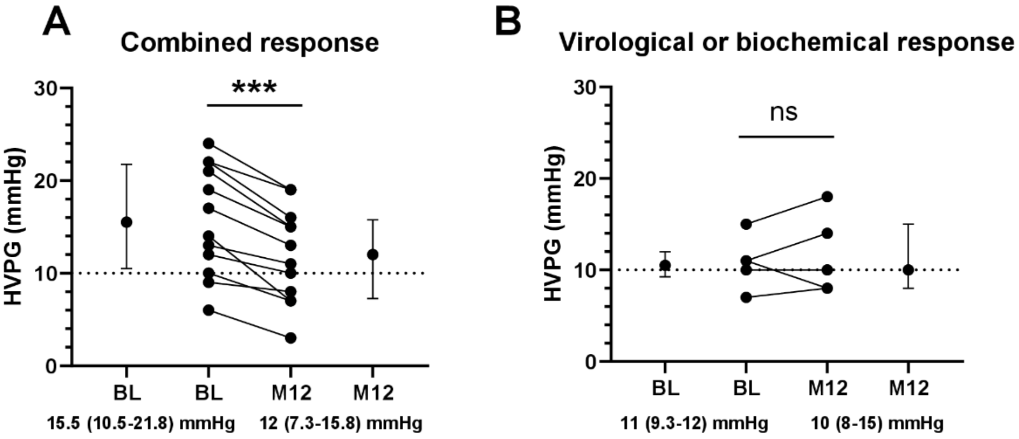

Supplementary figure 1: Comparison of HVPG at baseline and M12 of patients with combined (A) or with virological/biochemical (B) response. Medians with interquartile range and individual values are depicted. Wilcoxon signed rank test was used to compare baseline and M12 results.

*** $p < 0.001$; ns, not significant

Table S1.

HVPG (mmHg)		Responder	Non-responder	p-value
CR	BL	15.5 (10.5-21.8)	11 (10-14)	0.129
	M12	12 (7.3-15.8)	10 (8.5-17)	0.923
	Median HVPG change (BL-M12) [mmHg]	-3.5 (-5.75 - -2.25)	0.5 (-0.75 – 3.0)	<0.001
VR	BL	14.5 (10-21.3)	11 (9.3-12.8)	0.172
	M12	12 (7.8-16.5)	10 (8-15.8)	0.828
	Median HVPG change (BL-M12) [mmHg]	-3 (-5.25 - -1.75)	0.5 (-1.5 – 3.0)	0.012
BR	BL	12.5 (10-20.5)	13 (10.3-17.3)	0.950
	M12	10.5 (8-15)	14 (10-20.3)	0.272
	Median HVPG change (BL-M12) [mmHg]	-3.0 (-4.75 - -1.25)	1.5 (-0.75 – 3.0)	0.013

Table S1. Comparison of baseline and on-treatment HVPG values stratified by treatment response. Median with interquartile range for continuous parameters is depicted, Mann Whitney U test was used to compare groups.

Abbreviations: BL, baseline; BR, biochemical response; CR, combined response; HVPG, hepatic venous pressure gradient; M12, minimum of 12 months of antiviral treatment; VR, virological response

Table S2.

**LSM (kPa)**		**Baseline**	**M12**	**p-value**
All patients		24 (13.5-28.5)	13.5 (9.0-22.7)	**<0.001**
CR	Yes	24.1 (13.9-31.2)	13.3 (9.6-22.1)	0.004
	No	20.8 (11-26)	14.4 (7.3-25)	0.039
VR	Yes	24.1 (15.7-30.7)	16.6 (10-23.9)	0.002
	No	16.2 (9.1-25.5)	10.5 (6.8-19.4)	0.156
BR	Yes	18.7 (12-28)	11.5 (8.8-18.7)	0.001
	No	25.8 (24.3-30)	23.9 (17.9-23.9)	0.250

Table S2. Comparison of baseline and on-treatment liver stiffness measurement results stratified by treatment response. Median with interquartile range for continuous parameters is depicted, Wilcoxon signed rank test was used to compare groups.

Abbreviations: BL, baseline; BR, biochemical response; CR, combined response; LSM, liver stiffness measurement; M12, minimum of 12 months of antiviral treatment; VR, virological response

Table S3.

LSM (kPa)		Responder	Non-responder	p-value
CR	BL	24.1 (13.9-31.2)	20.8 (11-26)	0.473
	M12	13.3 (9.6-22.1)	14.4 (7.3-25)	0.792
VR	BL	24.1 (15.7-30.7)	16.2 (9.1-25.5)	0.179
	M12	16.6 (10-23.9)	10.5 (6.8-19.4)	0.246
BR	BL	18.7 (12-28)	25.8 (24.3-30)	0.152
	M12	11.5 (8.8-18.7)	23.9 (17.9-23.9)	0.052

Table S3. Comparison of baseline and on-treatment liver stiffness measurement values stratified by treatment response. Median with interquartile range for continuous parameters is depicted, Mann Whitney U test was used to compare groups.

Abbreviations: BL, baseline; BR, biochemical response; CR, combined response; LSM, liver stiffness measurement; M12, minimum of 12 months of antiviral treatment; VR, virological response

Table S4.

	BL			M12		
	CSPH “ruled out”		CSPH “ruled in”	CSPH “ruled out”		CSPH “ruled in”
Total cohort: n = 20	LSM ≤ 15 kPa and PLT ≥ 150 G/L	unclassified	LSM ≥ 25 kPa	LSM ≤ 15 kPa and PLT ≥ 150 G/L	unclassified	LSM ≥ 25 kPa
Patients, n (%)	2 (10%)	11 (55%)	7 (35%)	1 (5%)	15 (75%)	4 (20%)
CSPH, n	1	9	7	0	10	4
Performance	NPV 50% Sensitivity 94%		PPV 100% Specificity 100%	NPV 100% Sensitivity 100%		PPV 100% Specificity 100%

Table S4. Diagnostic performance of the Baveno VII criteria at baseline and after a minimum treatment duration of 12 months.

Abbreviations: CSPH, clinically significant portal hypertension; HVPG, hepatic venous pressure gradient; LSM, liver stiffness measurement; M12, minimum of 12 months of antiviral treatment; NIT, non-invasive test

Table S5.

		Baseline	M12	p-value
**LBP (ng/mL)**				
All patients		3477 (2384-4241)	3265 (2108-4472)	0.623
Combined response	Yes	3688 (2939-(4241)	3545 (2513-4638)	0.765
	No	2805 (2060-4683)	2611 (1684-4357)	0.383
Virological response	Yes	3477 (2404-4121)	3265 (2324-4507)	0.735
	No	3477 (2141-5156)	3384 (1699-5271)	0.563
Biochemical response	Yes	3688 (2514-4543)	6545 (2108-4348)	0.847
	No	2805 (1739-3598)	2611 (1510-3682)	0.625
**sCD163 (ng/mL)**				
All patients		**1813 (1315-2063)**	**1405 (1041-1965)**	**0.003**
Combined response	**Yes**	**1865 (1629-2609)**	**1405 (1049-1965)**	**0.001**
	No	1223 (1100-2004)	1367 (1041-1968)	0.313
Virological response	**Yes**	**1865 (1615-2094)**	**1405 (1029-1832)**	**<0.001**
	No	1199 (1077-1934)	1403 (997-2157)	0.688
Biochemical response	**Yes**	**1841 (1587-2154)**	**1405 (1021-1965)**	**<0.001**
	No	1223 (1112-1858)	1367 (1099-2063)	1.0
**TGFb (pg/mL)**				
All patients		18719 (12025-24968)	18587 (14483-23160)	0.324
Combined response	Yes	19926 (16537-24968)	18587 (14596-24569)	0.515
	No	14848 (10585-26292)	18482 (11810-23160)	0.641
Virological response	Yes	18719 (12486-23480)	17584 (14170-23152)	0.903
	No	19509 (9619-28974)	22209 (13677-24931)	0.844
	Yes	18718 (12970-22562)	20117 (14596-23160)	0.528

Biochemical response	No	20214 (11860-31502)	15511 (11810-26598)	1.0
**PDGF (pg/mL)**				
All patients		138.4 (56.4-330.8)	152.9 (46.7-263.9)	0.475
Combined response	Yes	125.5 (51.1-295)	109.6 (28.2-224.2)	0.301
	No	207.6 (72.6-374.6)	210.7 (86.6-278.5)	0.945
Virological response	Yes	115.7 (53.7-264.5)	109.6 (38.2-229.8)	0.542
	No	327.9 (101.1-395.1)	231 (118.9-354.4)	0.844
Biochemical response	Yes	138.4 (51.1-303.2)	143.5 (34.1-262.7)	0.274
	No	249.4 (72.6-435.6)	210.7 (96.6-491.6)	0.875
**Ang1 (pg/mL)**				
All patients		15176 (8272-20352)	14099 (8938-19913)	0.245
Combined response	Yes	15176 (10928-19596)	13074 (9674-19913)	0.233
	No	13218 (6745-20762)	14303 (7116-22836)	0.844
Virological response	Yes	15020 (9256-19171)	12348 (9182-19641)	0.426
	No	19595 (5838-22063)	17065 (7547-24339)	0.563
Biochemical response	Yes	15176 (9942-19596)	15028 (9674-19913)	0.298
	No	14157 (6849-24438)	10568 (7077-21455)	0.875
**Ang2 (pg/mL)**				
All patients		**2793 (1870-3604)**	**2527 (1379-3135)**	**0.030**
Combined response	**Yes**	**3542 (1955-3815)**	**2227 (1424-3377)**	**0.001**
	No	2423 (1581-2925)	2607 (1284-3080)	0.945
Virological response	**Yes**	**3250 (1911-3678)**	**2227 (1462-3142)**	**<0.001**
	No	2423 (1478-3159)	2613 (1197-3997)	0.688
	**Yes**	**3159 (1955-3815)**	**2527 (1424-3135)**	**0.013**

Biochemical response	No	2326 (1541-2925)	2147 (1241-5552)	0.875
**Ang2/Ang1 ratio**				
All patients		0.22 (0.10-0.35)	0.18 (0.08-0.35)	0.179
Combined response	Yes	0.21 (0.10-0.35)	0.18 (0.08-0.32)	0.266
	No	0.22 (0.09-0.41)	0.16 (0.08-0.39)	0.461
Virological response	Yes	0.24 (0.10-0.38)	0.18 (0.09-0.36)	0.104
	No	0.17 (0.07-0.77)	0.16 (0.06-0.47)	0.844
Biochemical response	Yes	0.22 (0.10-0.35)	0.18 (0.08-0.32)	0.106
	No	0.19 (0.08-0.40)	0.26 (0.06-0.73)	0.875
**VEGF (pg/mL)**				
All patients		34.6 (34.6-813.6)	34.6 (34.6-4671.5)	0.074
Combined response	Yes	34.6 (34.6-618.8)	34.6 (34.6-5064.9)	0.313
	No	34.6 (34.6-1661.8)	34.6 (34.6-4242.5)	0.250
Virological response	Yes	34.6 (34.6-2214)	34.6 (34.6-5355.9)	0.219
	No	34.6 (34.6-441.4)	34.6 (34.6-3384)	0.500
Biochemical response	Yes	34.6 (34.6-34.6)	34.6 (34.6-4671.5)	0.156
	No	1661.8 (34.6-5180.8)	1495 (34.6-5885.6)	0.500

Table S5. Comparison of PH-related biomarkers reflecting bacterial translocation, macrophage activation, (dys)angiogenesis and fibrogenesis. Wilcoxon signed rank test was used to compare baseline and M12 parameters. Abbreviations: Ang1, Angiopoietin-1; Ang2, Angiopoietin-2; LBP, lipopolysaccharide-binding protein; M12, minimum of 12 months of antiviral treatment; PDGF, platelet-derived growth factor; TGFb, transforming growth factor beta; VEGF, vascular endothelial growth factor

Table S6.

		Responder	Non-responder	p-value
**LBP (ng/mL)**				
CR	BL	3688 (2939-(4241)	2805 (2060-4683)	0.343
	M12	3545 (2513-4638)	2611 (1684-4357)	0.473
VR	BL	3477 (2404-4121)	3477 (2141-5156)	0.968
	M12	3265 (2324-4507)	3384 (1699-5271)	0.904
BR	BL	3688 (2514-4543)	2805 (1739-3598)	0.178
	M12	6545 (2108-4348)	2611 (1510-3682)	0.335
**sCD163 (ng/mL)**				
CR	BL	1865 (1629-2609)	1223 (1100-2004)	0.069
	M12	1405 (1049-1965)	1367 (1041-1968)	0.910
VR	BL	1865 (1615-2094)	1199 (1077-1934)	0.062
	M12	1405 (1029-1832)	1403 (997-2157)	0.904
BR	BL	1841 (1587-2154)	1223 (1112-1858)	0.178
	M12	1405 (1021-1965)	1367 (1099-2063)	0.820
**TGFb (pg/mL)**				
CR	BL	19926 (16537-24968)	14848 (10585-26292)	0.521
	M12	18587 (14596-24569)	18482 (11810-23160)	0.734
VR	BL	18719 (12486-23480)	19509 (9619-28974)	0.904
	M12	17584 (14170-23152)	22209 (13677-24931)	0.602
BR	BL	18718 (12970-22562)	20214 (11860-31502)	0.617
	M12	20117 (14596-23160)	15511 (11810-26598)	0.554
**PDGF (pg/mL)**				
CR	BL	125.5 (51.1-295)	207.6 (72.6-374.6)	0.521

	M12	109.6 (28.2-224.2)	210.7 (86.6-278.5)	0.176
VR	BL	115.7 (53.7-264.5)	327.9 (101.1-395.1)	0.207
	M12	109.6 (38.2-229.8)	231 (118.9-354.4)	0.173
BR	BL	138.4 (51.1-303.2)	249.4 (72.6-435.6)	0.437
	M12	143.5 (34.1-262.7)	210.7 (96.6-491.6)	0.283
**Ang1 (pg/mL)**				
CR	BL	15176 (10928-19596)	13218 (6745-20762)	0.678
	M12	13074 (9674-19913)	14303 (7116-22836)	0.910
VR	BL	15020 (9256-19171)	19595 (5838-22063)	0.602
	M12	12348 (9182-19641)	17065 (7547-24339)	0.602
BR	BL	15176 (9942-19596)	14157 (6849-24438)	0.936
	M12	15028 (9674-19913)	10568 (7077-21455)	0.494
**Ang2 (pg/mL)**				
CR	BL	3542 (1955-3815)	2423 (1581-2925)	0.246
	M12	2227 (1424-3377)	2607 (1284-3080)	0.910
VR	BL	3250 (1911-3678)	2423 (1478-3159)	0.363
	M12	2227 (1462-3142)	2613 (1197-3997)	1.0
BR	BL	3159 (1955-3815)	2326 (1541-2925)	0.242
	M12	2527 (1424-3135)	2147 (1241-5552)	0.892
**Ang2/Ang 1 ratio**				
CR	BL	0.21 (0.10-0.35)	0.22 (0.09-0.41)	0.970
	M12	0.18 (0.08-0.32)	0.16 (0.08-0.39)	0.970
VR	BL	0.24 (0.10-0.38)	0.17 (0.07-0.77)	0.602
	M12	0.18 (0.09-0.36)	0.16 (0.06-0.47)	0.904
BR	BL	0.22 (0.10-0.35)	0.19 (0.08-0.40)	0.750

	M12	0.18 (0.08-0.32)	0.26 (0.06-0.73)	0.820
**VEGF (pg/mL)**				
CR	BL	34.6 (34.6-618.8)	34.6 (34.6-1661.8)	0.881
	M12	34.6 (34.6-5064.9)	34.6 (34.6-4242.5)	0.900
VR	BL	34.6 (34.6-2214)	34.6 (34.6-441.4)	0.447
	M12	34.6 (34.6-5355.9)	34.6 (34.6-3384)	0.524
BR	BL	34.6 (34.6-34.6)	1661.8 (34.6-5180.8)	0.097
	M12	34.6 (34.6-4671.5)	1495 (34.6-5885.6)	0.645

Table S6. Comparison of baseline and on-treatment biomarkers stratified by treatment response. Median with interquartile range for continuous parameters is depicted, Mann Whitney U test was used to compare groups.

Abbreviations: Ang1, Angiopoietin-1; Ang2, Angiopoietin-2; BL, baseline; BR, biochemical response; CR, combined response; LBP, lipopolysaccharide-binding protein; M12, minimum of 12 months of antiviral treatment; PDGF, platelet-derived growth factor; TGFb, transforming growth factor beta; VEGF, vascular endothelial growth factor; VR, virological response

Table S7.

	HVPG decline ≥10%<br>n = 13		p-value	No or < 10 % HVPG decline<br>n = 7		p-value
	Baseline	M12		Baseline	M12	
LBP (ng/mL)	3779 (3063-4462)	3717 (2593-4854)	0.910	2374 (2020-3719)	2434 (1677-3980)	0.078
sCD163 (ng/mL)	**1874 (1687-2222)**	**1431 (1088-2076)**	**0.001**	1204 (1081-1826)	1290 (1035-1515)	0.469
TGF beta (pg/mL)	19654 (16580-24224)	18966 (14989-24221)	0.735	12717 (10255-27711)	15696 (10639-23149)	0.938
PDGF (pg/mL)	133.3 (53.8-279.7)	80.4 (27.1-219.8)	0.191	282.0 (55.5-374.8)	233.5 (148.2-280.1)	0.813
Ang 1 (pg/mL)	15240 (11331-20323)	14825 (10044-19777)	0.146	7781 (6538-20533)	13373 (6901-24150)	0.938
Ang 2 (pg/mL)	**3551 (1979-4055)**	**2630 (1510-3259)**	**0.001**	2061 (1492-2803)	2424 (1211-3172)	0.578
Ang 2/Ang 1 ratio	0.20 (0.10-0.35)	0.18 (0.08-0.29)	0.216	0.24 (0.08-0.45)	0.13 (0.06-0.41)	0.578
VEGF (pg/mL)	34.6 (34.6-424.1)	34.6 (34.6-3876.6)	0.313	34.6 (34.6-2541.6)	34.6 (34.6-4671.5)	0.250

Table S7. Comparison of markers reflecting bacterial translocation, macrophage activation, neoangiogenesis and fibrogenesis in patients with and without ≥ 10% HVPG decline. Wilcoxon signed rank test was used to compare baseline and M12 parameters.

Abbreviations: Ang1, Angiopoietin-1; Ang2, Angiopoietin-2; LBP, lipopolysaccharide-binding protein; M12, minimum of 12 months of antiviral treatment; PDGF, platelet-derived growth factor; TGFb, transforming growth factor beta; VEGF, vascular endothelial growth factor

Table S8.

		**Baseline**	**M12**	**p-value**
**CRP (mg/l)**				
All patients		1.4 (0.65-2.48)	0.75 (0.6-3.55)	0.396
CR	Yes	2.1 (0.93-3.25)	0.65 (0.6-3.55)	0.140
	No	0.85 (0.6-1.5)	0.85 (0.6-3.83)	0.750
VR	Yes	1.95 (0.75-2.75)	0.65 (0.6-3.45)	0.140
	No	1.05 (0.58-2)	1.05 (0.6-10.4)	0.750
BR	Yes	1.95 (0.93-3.03)	0.8 (0.6-3.55)	0.260
	No	0.7 (0.53-0.88)	0.7 (0.6-3.73)	1.0
**WBC (x1000/µl)**				
All patients		3.6 (2.6-4.2)	3.2 (2.2-4.4)	0.280
CR	Yes	3.55 (2.7-4.3)	3.2 (2.4-3.8)	0.077
	No	3.7 (2.5-4.2)	3.6 (2.1-4.7)	0.570
VR	Yes	3.6 (2.5-4.2)	3.2 (2.2-3.9)	0.093
	Yes	3.7(2.6-4.7)	3.8 (2.2-5.2)	0.688
BR	Yes	3.6 (2.6-4.3)	3.2 (2.4-4.1)	0.298
	No	3.7 (2.3-4.2)	3.2 (1.9-4.6)	1.0

Table S8. Comparison of baseline and on-treatment levels of c-reactive protein and white blood cell counts stratified by treatment response. Median with interquartile range for continuous parameters is depicted, Wilcoxon signed rank test was used to compare groups.

Abbreviations: BL, baseline; BR, biochemical response; CR, combined response; CRP, c-reactive protein; M12, minimum of 12 months of antiviral treatment; VR, virological response; WBC, white blood cells
